# Signal hierarchical petri nets: Formal semantics of hierarchical regulatory control of biological systems

**DOI:** 10.1371/journal.pone.0355540

**Published:** 2026-08-21

**Authors:** Eugênio Simão

**Affiliations:** Department of Computer Science, Universidade Federal de Santa Catarina (UFSC), Araranguá, Santa Catarina, Brazil; Indian Institute of Technology Madras, INDIA

## Abstract

Cellular commitment thresholds—such as metabolite concentrations triggering irreversible developmental transitions—cannot be derived as structural properties of network topology in classical computational formalisms. Existing numerical approaches (e.g., ODE bifurcation analysis) predict thresholds only after full parameterization; the threshold is an output of fitting, not of topology. The fundamental reason: classical models treat metabolites either as passive substrates (metabolic networks) or as Boolean switches (gene networks), lacking unified semantics for molecules functioning simultaneously as metabolic currencies and regulatory signals. We present signal hierarchy theory establishing formal foundations for hierarchical biological control. The theory proves two structural theorems: signal flow graphs must be acyclic, and lower-layer signal depletion structurally preempts higher-layer transitions—formalizing that resource exhaustion overrides regulatory programs regardless of controller abundance. We implement this theory as signal hierarchical Petri nets (SHPN), extending classical Bio-PN from 5-tuple to 13-tuple formalism with signal flow arcs enabling consumptive information propagation. Modelers designate any metabolite as signal place (ATP, GTP, NADH, cAMP, Ca^2+^) when biological evidence supports regulatory gating. Unlike test arcs (non-consuming catalysis), signal flow arcs create basin boundaries with commitment threshold $M_{\text{commit}}=\theta +W_{s}$ computable directly from arc parameters—no simulation, no fitting. The formalism unifies metabolic-regulatory modeling: normal arcs for horizontal mass transfer, signal flow arcs for vertical information propagation. Applied to *Bacillus subtilis* sporulation, the formalism captures the Fujita and Losick (2005) observation: abrupt induction of Spo0A* yields only ≈5% sporulation while gradual KinA phosphorelay accumulation yields ≈52%. The asymmetry emerges structurally from the $\sigma_{H}$ commitment separatrix (≈1.60μM): gradual accumulation allows the $\sigma_{H}$ positive-feedback loop to cross threshold stochastically; an abrupt pulse fires before $\left[\sigma_{H}\right]$ builds. Stochastic sweep simulation (25 conditions, 50 replicates) reproduces the 52% vs. ≈5% contrast; all arc parameters (Γ, phosphorelay rates, $\sigma_{H}$ feedback) derive from published biological measurements—none was optimized to match this ratio.

## 1. Introduction

Cells make irreversible commitment decisions based on metabolite availability. When bacteria enter sporulation, cancer cells commit to division, or neurons release neurotransmitters, these transitions occur at specific molecular thresholds. Yet classical formalisms cannot derive these thresholds as structural properties of network topology—they require global parameter fitting or numerical simulation, so the threshold is an output of data, not of network structure.

### 1.1. The threshold prediction problem

Classical approaches fail at different levels. Flux Balance Analysis (FBA) optimizes metabolic flux distributions but lacks regulatory control semantics [1]. Boolean network models capture genetic logic but treat metabolites as external parameters rather than dynamic variables [2]. Classical hybrid Petri nets separate metabolism (continuous dynamics) and regulation (discrete logic) into distinct sub-models without unified semantics for signal designation [3,4]. Biological Petri nets [5–7] with test arcs model catalytic regulation but cannot express signal consumption—the irreversible depletion creating commitment finality [8,9].

The expressiveness gap is fundamental: no existing Petri net formalism provides semantics for molecules that simultaneously transfer mass (metabolism) and broadcast information (regulation) through modeler designation, making threshold computation a structural rather than parametric property. Metabolites designated as regulatory signals must participate in biochemical transformations (normal arcs, mass transfer) and control pathway activation (regulatory arcs, information transfer) within unified model semantics. Classical approaches force separation: FBA ignores regulation, Boolean networks parameterize metabolites, hybrid Petri nets partition concerns into distinct sub-models. Without unified semantics enabling dual participation through signal type assignment, threshold values emerge only through experimental measurement or parameter fitting, never through topology-driven threshold computation.

### 1.2. Contributions

**Fig 1:**
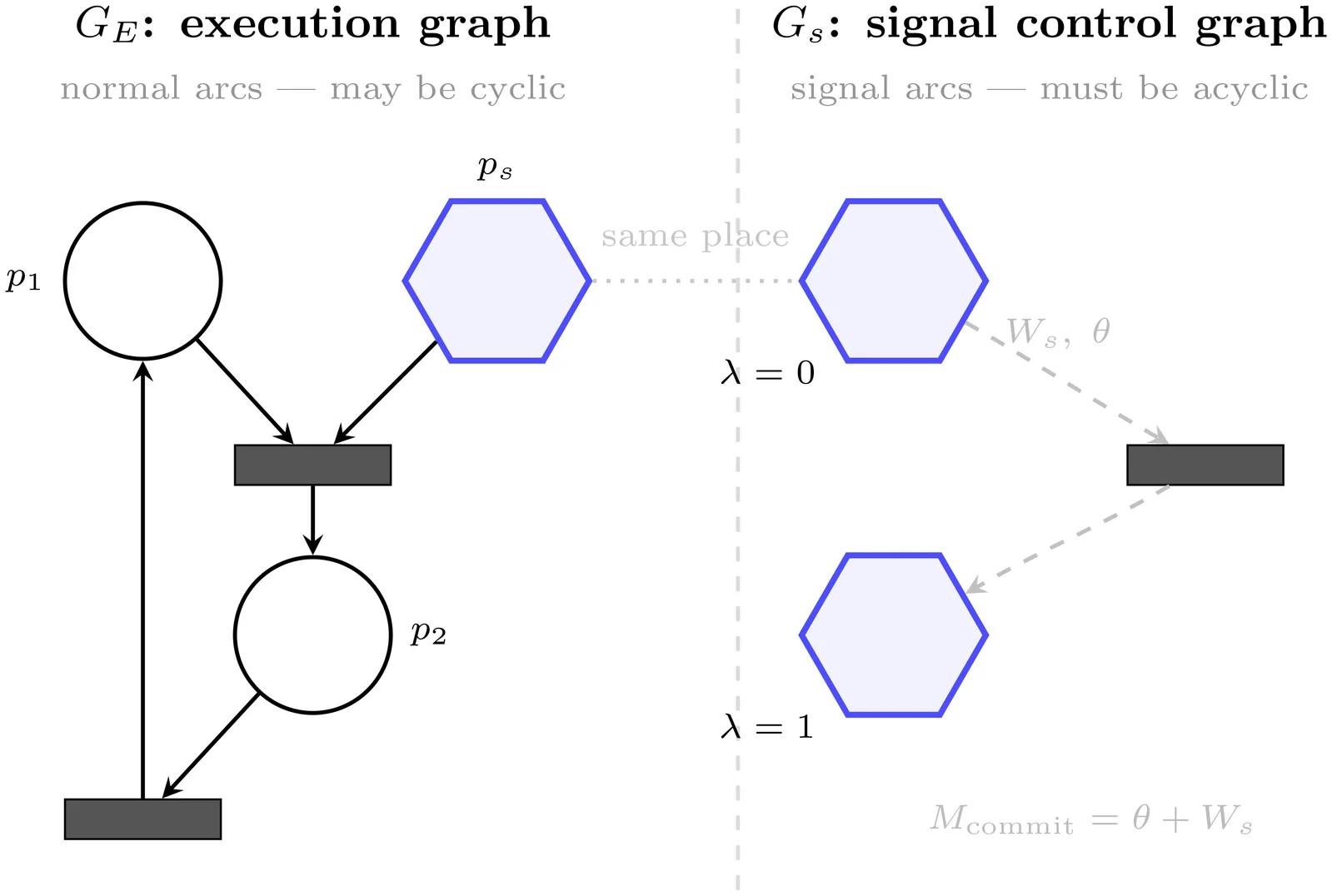
Dual-graph architecture of SHPN. The execution graph $G_{E}$ (left) carries stoichiometric mass transfer through normal arcs (solid black) and may contain cycles. Signal places (blue hexagons, $p_{s}$) participate in both graphs simultaneously. The signal control graph $G_{s}=(\Psi ,F_{s})$ (right) extracts those places into a directed acyclic graph (POSet); dashed arcs carry signal weight $W_{s}$ and basin-floor threshold θ, giving the commitment criterion $M_{commit}=\theta +W_{s}$. Layer depth λ encodes the hierarchy of commitment decisions. Acyclicity of $G_{s}$ formalizes biological irreversibility: a committed cell cannot uncommit through the same hierarchical channel. Cycles in $G_{E}$ remain fully unrestricted.

This work presents signal hierarchy theory and its computational realization as signal hierarchical Petri nets, enabling topology-driven threshold computation through four original contributions:

1. **Signal Hierarchy Theory:** We establish formal foundations for hierarchical biological control proving two structural theorems. The acyclicity theorem demonstrates that connected signal flow graphs (topology-explicit $F_{s}$ arcs defining commitment structure) must be directed acyclic graphs—formalizing commitment finality: once a decision is made, the cell must follow that biochemical path irreversibly without alternative routes back. The preemption theorem proves that lower-layer signal depletion structurally disables higher-layer transitions regardless of higher-layer token abundance—capturing the principle that resource exhaustion overrides regulatory programs. Critically, acyclicity ensures one-way commitment trajectories through connected channels, while feedback regulation occurs abundantly through remote sensing (rate functions Φ; formally defined in Section 3.2) without creating reversible commitment paths.
2. **Signal Hierarchical Control**: We introduce signal control theory establishing a graph of information layered over the graph of execution (Fig 1). By assigning signal type to selected places and arcs, we create vertical information channels broadcasting mass availability from Layer 0 (executive metabolic layer) to higher regulatory layers, while horizontal execution proceeds through stoichiometric transformations. Signal flow arcs implement consumptive commitment semantics distinct from test arcs (non-consuming catalysis): token consumption creates irreversible decisions partitioning state space through basin boundaries. Layer functions assign hierarchical organization, and preemption predicates formalize vertical dependencies where lower-layer depletion structurally disables higher-layer transitions.
3. **Signal Hierarchical Petri Nets**: We extend classical Bio-PN [10,11] from 5-tuple to 13-tuple hybrid formalism incorporating signal places (Ψ), signal flow arcs ($F_{s}$), signal arc weights ($W_{s}$), layer functions (λ), and enablement thresholds (θ). SHPN is a hybrid Petri net in the sense of supporting coexistent continuous and stochastic dynamics [4,12], but extends classical hybrid PN by adding unified signal-type semantics that resolve the sub-model separation limitation of prior approaches [3]: rather than partitioning metabolism and regulation into distinct sub-models, SHPN allows modeler-designated places to participate in both simultaneously. The formalism therefore supports both deterministic and stochastic simulation engines [13] while providing unified semantics for metabolic-regulatory integration. The extension preserves Bio-PN semantics while adding hierarchical capabilities: normal arcs for horizontal mass transfer, signal flow arcs for vertical information propagation. Modelers designate any metabolite (ATP, GTP, NADH, cAMP, Ca^2+^) as signal place $p_{s}\in \Psi$ when biological evidence supports regulatory gating role. Designated signals participate through both arc types simultaneously, unifying metabolic and regulatory roles within a single hybrid model.
4. **Metabolic-Regulatory Unified Modeling:** By unifying metabolic and regulatory networks through signal type assignment, commitment thresholds emerge from topology as structural properties computable via $M_{\text{commit}}=\theta (t)+W_{s}((p_{s},t))$. Applied to *B. subtilis* sporulation, the formalism provides a mechanistic account of the Fujita and Losick [14] timing-dependent outcome asymmetry (abrupt ≈5%, gradual ≈52%): the $\sigma_{H}$ effective commitment threshold at ≈1.60μM is identified empirically via simulation (Proposition 5), and only the gradual phosphorelay accumulation allows $\left[\sigma_{H}\right]$ to ramp across it. This mechanistic prediction—inexpressible in classical Bio-PN lacking Γ-threshold semantics—is quantified in Section 6.

**Validation:** Mathematical validation through proven theorems establishes correctness. Biological validation through *B. subtilis* case study demonstrates practical applicability, following established methods for Petri net pathway validation [15–17]. Together, these demonstrate that SHPN provides a structural account of biological commitment that is testable against independent experimental data and does not require global parameter fitting to reproduce experimental outcome ratios.

## 2. Signal hierarchy theory

We establish formal foundations for hierarchical biological control systems by proving structural properties of signal flow networks [5,6]. The theory introduces three conceptual innovations enabling threshold predictability: (1) signal flow graphs as directed acyclic graphs enforcing hierarchical organization, (2) preemption semantics where lower-layer depletion disables higher-layer transitions, and (3) basin boundaries computable from network topology without simulation.

### 2.1. Fundamental concepts

**Definition 1** (Signal Flow Graph). *Let T be the set of transitions of the underlying Petri net. A signal flow graph* $G_{s}=(\Psi ,F_{s})$ *consists of signal places* Ψ⊆P *(information channels) and signal flow arcs* $F_{s}\subseteq (\Psi \times T)\cup (T\times \Psi )$ *representing consumptive information propagation. Unlike metabolic arcs transferring mass, signal flow arcs transfer regulatory information consumed during transition firing.*

**Definition 2** (Layer Function). *A layer function* $\lambda :\Psi \to {\mathbb{N}}_{0}$ *assigns hierarchical depth to signal places via topological sort of* $G_{s}$*. For all signal flow edges, we enforce:*


$$\begin{array}{c} \left(p_{i},t)\in F_{s}\wedge (t,p_{j})\in F_{s}⟹\lambda (p_{i})<\lambda (p_{j}\right) \end{array}$$

*establishing directed information flow from lower (metabolic) to higher (regulatory) layers.*

**Definition 3** (Basin Boundary). *For transition t with signal input arc* $(p_{s},t)\in F_{s}$*, the commitment marking*


$$\begin{array}{c} M_{commit}(p_{s})=\theta (t)+W_{s}((p_{s},t)) \end{array}$$

*defines basin boundary partitioning reachable state space:*

- ***Below boundary:*** $B_{below}=\{M:M(p_{s})<M_{commit}(p_{s})\}$ *(transition t disabled)*
- ***At boundary:*** $B_{boundary}=\{M:M(p_{s})=M_{commit}(p_{s})\}$ *(firing consumes* $W_{s}$*, leaves residual* $M^{\prime }(p_{s})=\theta (t)$)
- ***Above boundary:*** $B_{above}=\{M:M(p_{s})>M_{commit}(p_{s})\}$ *(firing optional)*

*Firing from* $M\in B_{boundary}$ *produces* $M^{\prime }\in B_{below}\cup B_{boundary}$ *when* $M^{\prime }(p_{s})=\theta (t)\leq M_{commit}(p_{s})$*, creating one-way barrier: no firing sequence from B*_*below*_ *reaches B*_*above*_ *after signal consumption.*

### 2.2. Structural theorems

**Theorem 1 (Acyclicity Requirement).** *Signal flow graphs must be directed acyclic graphs (DAGs). Formally:* $G_{s}=(\Psi ,F_{s})$ *admits valid layer function* $\lambda :\Psi \to {\mathbb{N}}_{0}$ *satisfying* $\lambda (p_{i})<\lambda (p_{j})$ *for all paths* $p_{i}\overset{t\;}{\to }p_{j}$ *if and only if* $G_{s}$ *is acyclic.*

*Proof.* (⇒) Assume valid layer function λ exists satisfying $\lambda (p_{i})<\lambda (p_{j})$ for all edges $\left(p_{i},t,p_{j}\right)$ where *t* connects $p_{i}$ to $p_{j}$ via signal flow arcs. Suppose for contradiction that $G_{s}$ contains cycle


$$\begin{array}{c} p_{1}\overset{t_{1}\;}{\to }p_{2}\overset{t_{2}\;}{\to }\cdots \overset{t_{n}\;}{\to }p_{1}. \end{array}$$

By layer function constraint applied to each edge:


$$\begin{array}{c} \lambda (p_{1})<\lambda (p_{2})<\lambda (p_{3})<\cdots <\lambda (p_{n})<\lambda (p_{1}) \end{array}$$

Combining inequalities yields $\lambda (p_{1})<\lambda (p_{1})$, contradicting irreflexivity of < on ${\mathbb{N}}_{0}$. Therefore $G_{s}$ must be acyclic.

(⇐) Assume $G_{s}$ is acyclic. We construct valid λ via topological sort algorithm:

1. Initialize λ(p)=0 for all p∈Ψ
2. While exist unprocessed places: select *p* with indegree 0 in remaining graph
3. For each outgoing edge $\left(p,t,p^{\prime }\right)$: set $\lambda (p^{\prime })=\max(\lambda (p^{\prime }),\lambda (p)+1)$
4. Remove *p* from graph, update indegrees

Algorithm terminates because $G_{s}$ is finite and acyclic (no place depends on itself). For any edge $\left(p_{i},t,p_{j}\right)$, topological ordering ensures $p_{i}$ processed before $p_{j}$, thus $\lambda (p_{i})<\lambda (p_{j})$ by construction. Hence valid layer function exists. □

**Biological significance:** Acyclicity ensures commitment finality—once a decision is made through connected hierarchical channels, there is no alternative path back. When a cell commits to sporulation, $\sigma_{H}$ rises above its effective kinetic threshold $\theta_{\sigma H}=1.60\;\mu$M (via the rate function $\Phi (T_{\text{septation}})\propto [\sigma_{H}{]}^{2}$), enabling *T*_septation_ to fire and consume GTP tokens irreversibly via the GTP_pool signal-flow arc—it must then follow that biochemical program irreversibly. The cell cannot “uncommit” and return to vegetative growth through the same signal flow path that triggered commitment. However, feedback regulation occurs abundantly through remote sensing (see Section 3.2): transcription factors can sense metabolite concentrations via rate functions Φ without creating reversible commitment paths. The acyclicity constraint applies to topology-explicit connected paths (signal flow arcs $F_{s}$ defining irreversible commitment structure), not to equation-implicit remote observations (rate functions Φ enabling feedback without reversibility). Signal consumption creates one-way state transitions: firing from basin boundary $M_{commit}$ depletes signals, making reverse traversal impossible. This formalizes biological commitment: development proceeds forward through irreversible decisions, while metabolic feedback maintains homeostasis through remote sensing.

**Theorem 2** (Hierarchical Preemption). *Lower-layer signal depletion structurally disables higher-layer transitions, assuming the PreemptionCheck predicate is enforced: transition* $t_{j}$ *may fire only if every transition* $t_{i}$ *that produces its signal inputs via* $F_{s}$ *paths satisfies its local enablement conditions (NormalEnabled, TestEnabled, and SignalEnabled). Formally: if* $\lambda (p_{i})<\lambda (p_{j})$ *and there exists connected path* $p_{i}⇝p_{j}$ *through signal flow arcs, then*

$M(p_{i})<\theta (t_{i})\;for\;any\;t_{i}\in {\;}^{•}p_{j}⟹\neg \;Enabled\;(t_{j},M)\;for\;all\;t_{j}\in p_{j}^{•},$

*regardless of* $M(p_{j})$.

*Proof.* We prove by induction on layer depth $k=\lambda (p_{j})-\lambda (p_{i})$.

**Base case** (*k* = 1): Places $p_{i},p_{j}$ in adjacent layers with $\lambda (p_{i})=\ell$ and $\lambda (p_{j})=\ell +1$. By layer function definition, there exists $(p_{i},t_{i})\in F_{s}$ and $(t_{i},p_{j})\in F_{s}$. The SignalEnabled predicate for $t_{i}$ requires $M(p_{i})\geq \theta (t_{i})+W_{s}((p_{i},t_{i}))$. If $M(p_{i})<\theta (t_{i})$, then since $W_{s}((p_{i},t_{i}))>0$, $M(p_{i})<\theta (t_{i})+W_{s}((p_{i},t_{i}))$, hence $\neg \text{SignalEnabled}(t_{i},M)$. By definition, $\text{PreemptionCheck}(t_{j},M)$ requires $\text{SignalEnabled}(t_{i},M)$ for every $t_{i}\in {\;}_{s}^{•}p_{s}$ where $p_{s}\in {\;}_{s}^{•}t_{j}$. Since $\neg \text{SignalEnabled}(t_{i},M)$, we have $\neg \text{PreemptionCheck}(t_{j},M)$ for all $t_{j}\in p_{j}^{•}$. Hence $\neg \text{Enabled}(t_{j},M)$.

**Inductive step**: Assume true for all layer gaps $k^{\prime }<k$. Consider $p_{i},p_{j}$ with $\lambda (p_{j})-\lambda (p_{i})=k>1$. There exists intermediate place $p_{m}$ with $\lambda (p_{i})<\lambda (p_{m})<\lambda (p_{j})$ on path $p_{i}⇝p_{m}⇝p_{j}$. By inductive hypothesis applied to $\left(p_{i},p_{m}\right)$ with gap $\lambda (p_{m})-\lambda (p_{i})<k$: if $M(p_{i})<\theta (t_{i})$, then $\neg \text{SignalEnabled}(t_{m},M)$ for all $t_{m}$ producing $p_{m}$ via $F_{s}$. Therefore $\neg \text{PreemptionCheck}(t_{j},M)$ for all $t_{j}\in p_{m}^{•}$ (direct consequence of ¬SignalEnabled of $p_{m}$’s producers). By inductive hypothesis applied to $\left(p_{m},p_{j}\right)$: this propagates ¬SignalEnabled to all $F_{s}$-producers of $p_{j}$, hence $\neg \text{PreemptionCheck}(t_{j},M)$ for all $t_{j}\in p_{j}^{•}$. Transitive closure over the $F_{s}$-acyclic path completes the proof. □

**Biological significance**: $\sigma_{H}$ depletion below the commitment separatrix ($\theta_{\sigma H}=1.60\;\mu$M) structurally disables septation and all downstream execution layers, regardless of Spo0A~P abundance. A cell with saturating phosphorelay output but $\sigma_{H}$ below threshold cannot commit—the commitment gate overrides integration-layer readiness. This captures hierarchical override: Layer 2 preempts Layer 3 through threshold enforcement, preventing commitment without the required $\sigma_{H}$ signal.

### 2.3. Basin boundary computability

**Theorem 3** (Irreversible Basin Partition). *For signal place* $p_{s}\in \Psi$ *with signal flow arc* $(p_{s},t)\in F_{s}$*, assuming no other transition regenerates* $p_{s}$ *via* $F_{s}$ *(i.e., there is no* $t^{\prime }\text{≠}t$ *with* $(t^{\prime },p_{s})\in F_{s}$*), firing transition t from commitment marking* $M_{\text{commit}}(p_{s})=\theta (t)+W_{s}((p_{s},t))$ *creates a one-way barrier in the reachability graph. Formally, let:*


$$\begin{array}{cc} B_{above} & =\{M\in ℛ(M_{0}):M(p_{s})>M_{commit}(p_{s})\}\\ B_{boundary} & =\{M\in ℛ(M_{0}):M(p_{s})=M_{commit}(p_{s})\}\\ B_{below} & =\{M\in ℛ(M_{0}):M(p_{s})<M_{commit}(p_{s})\} \end{array}$$

*where* $ℛ(M_{0})$ *denotes reachable markings. Then firing t from any* $M\in B_{boundary}$ *produces* $M^{\prime }\in B_{below}\cup B_{boundary}$*, and no firing sequence from B*_*below*_ *produces a marking in B*_*above*_.

Proof. Consider marking $M\in B_{\text{boundary}}$ with $M(p_{s})=M_{\text{commit}}(p_{s})=\theta (t)+W_{s}((p_{s},t))$. By signal enablement, $M(p_{s})\geq \theta (t)+W_{s}((p_{s},t))$ holds with equality. Firing *t* consumes $W_{s}((p_{s},t))$ tokens from $p_{s}$ (by firing rule for signal places), producing:


$$\begin{array}{cc} M^{\prime }(p_{s}) & =M(p_{s})-W_{s}((p_{s},t))+W_{s}((t,p_{s}))\\ & =(\theta (t)+W_{s}((p_{s},t)))-W_{s}((p_{s},t))+W_{s}((t,p_{s}))\\ & =\theta (t)+W_{s}((t,p_{s})) \end{array}$$

If $W_{s}((t,p_{s}))=0$ (no backward signal flow), then $M^{\prime }(p_{s})=\theta (t)<M_{\text{commit}}(p_{s})$, placing $M^{\prime }\in B_{\text{below}}$.

For irreversibility, consider any $M\in B_{\text{below}}$ with $M(p_{s})<M_{\text{commit}}(p_{s})$. To reach B_above_, must increase $M(p_{s})$ by producing tokens through some transition $t^{\prime }$ with $(t^{\prime },p_{s})\in F_{s}$. By hypothesis, no such $t^{\prime }$ exists. Thus B_below_ is an absorbing set: once below the commitment threshold, no reachable firing sequence returns to B_above_. □

**Corollary 4** (Threshold Predictability). *Commitment thresholds are computable from network topology without simulation:*


$$\begin{array}{c} M_{commit}(p_{s})=\theta (t)+W_{s}((p_{s},t)) \end{array}$$

*where* θ(t) *and* $W_{s}$ *derive from biological rate constants and stoichiometry.*

Proof. Direct consequence of Basin Boundary Definition. The commitment marking $M_{\text{commit}}(p_{s})$ is defined as the minimal marking enabling transition t such that firing consumes exactly the decision quota, leaving residual $M^{\prime }(p_{s})=\theta (t)$ (the enablement threshold). By signal enablement condition SignalEnabled(*t,M*):


$$\begin{array}{c} M(p_{s})\geq \theta (t)+W_{s}((p_{s},t))⟺M(p_{s})\geq M_{\text{commit}}(p_{s}) \end{array}$$

The minimal such marking is $M_{\text{commit}}(p_{s})=\theta (t)+W_{s}((p_{s},t))$ obtained by taking equality. Both parameters are formalism specifications: θ(t) is assigned via threshold function $\theta :T\to {\mathbb{R}}^{\geq 0}$, and $W_{s}((p_{s},t))$ via signal weight function $W_{s}:F_{s}\to {\mathbb{R}}^{+}$. No simulation or state space exploration required—values obtained directly from model specification via arithmetic. □

This establishes threshold prediction as structural property of signal hierarchical networks. Unlike parameter fitting requiring experimental data, topology-based computation proceeds from formalism alone: identify signal input arc $\left(p_{s},t\right)$, extract threshold θ(t) and weight $W_{s}((p_{s},t))$, compute commitment marking. Below M_commit_, transitions remain disabled; above, firing becomes possible but non-mandatory; at boundary, firing creates irreversibility.

## 3. Signal hierarchical control

Signal hierarchical control introduces a *graph of information* layered over the *graph of execution*. The execution graph (*P*, *T*, *F*) models biochemical transformations through ordinary places, transitions, and arcs following mass-action dynamics. The information graph $\left(\Psi ,F_{s}\right)$ models regulatory architecture through signal places and signal flow arcs defining hierarchical dependencies. By assigning signal type to selected places and arcs, we create vertical information channels broadcasting mass availability from Layer 0 to higher regulatory layers, while horizontal execution proceeds through stoichiometric transformations. This dual-graph architecture enables formal specification of control mechanisms: layer functions assign hierarchical organization, enablement predicates formalize vertical dependencies, signal consumption creates commitment semantics, and preemption mechanisms enforce irreversible decisions through state space partitioning.

### 3.1. Signal consumption semantics

The information graph operates through consumptive signal semantics distinct from classical arc types. Signal flow arcs $F_{s}$ implement information propagation where token consumption at Layer *k* triggers enablement re-evaluation at layers ℓ>k. This differs from:

**Normal arcs** (*F*): Model horizontal mass transfer with stoichiometric conservation. Firing consumes *W*((*p*,*t*)) inputs, produces *W*((*t*,*p*)) outputs, preserving molecular species through Layer 0 transformations.

**Test arcs** ($F_{t}$): Model catalysis through non-consuming local observation. Require $M(p)>\tau_{t}((p,t))$ without token transfer, enabling rate acceleration via arc-connected catalysts. Test arcs are the appropriate model for catalysis on two independent grounds. First, they are the established formalism for read-only access in Petri nets (contextual/read arcs, [18]): they are semantically distinct from loops (*W*(*p*,*t*)=*W*(*t*,*p*)), which consume and regenerate the same quantity of tokens, because loops contribu*t*e *t*o the stoichiometric firing rule while test arcs leave the marking unchanged — a semantic difference independent of concurrency. Second, test arcs correctly model biological catalysts: enzymes that enable reactions without being consumed must not contribute to mass balance, which loops would violate by introducing a spurious consume-then-regenerate cycle in the stoichiometric record.

**Signal flow arcs** ($F_{s}$): Model vertical information broadcast with consumptive commitment. These arcs require marking $M(p_{s})\geq \theta (t)+W_{s}((p_{s},t))$ and consume decision quota $W_{s}((p_{s},t))$ upon firing. Unlike horizontal mass conservation (normal arcs) or local catalysis (test arcs), signal consumption creates irreversibility—tokens represent commitment resources depleted upon firing, establishing one-way state transitions partitioning reachability space. The arc semantics $M^{\prime }(p_{s})=M(p_{s})-W_{s}((p_{s},t))+W_{s}((t,p_{s}))$ couple token transfer with vertical broadcast to all layers $\ell >\lambda (p_{s})$.

The information graph thus layers hierarchical control over biochemical execution: signal places serve as interfaces where Layer 0 mass dynamics broadcast regulatory information vertically through consumptive arcs, while horizontal transformations proceed through conserved mass transfer.

### 3.2. Connected vs. Remote information access

The formalism distinguishes between topology-explicit (connected) and equation-implicit (remote) concentration information transfer:

**Connected channels (Signal flow arcs** $F_{s}$**):** Created by assigning type “signal” to normal arcs, coupling horizontal mass transfer with vertical information broadcast. Signal flow arcs $F_{s}$ are ordinary arcs $(p_{s},t)\in F$ to which we assign signal semantics: token transfer at Layer *k* (horizontal mass flow) triggers enablement re-evaluation at layers ℓ>k (vertical information broadcast). Signal places $p_{s}\in \Psi$ function as information hubs through this type assignment: the same places receive mass via metabolic reactions (normal arcs *F*) and broadcast availability via signal flow arcs $F_{s}$. This creates an interface from Layer 0 (executive metabolic layer) to higher regulatory layers—mass flows horizontally through biochemical transformations, information about mass availability broadcasts vertically to the cellular regulatory environment. Arc topology makes regulatory dependencies explicit—connected paths define hierarchical control flow.

Test arcs $F_{t}$ implement non-consumptive observation of arc-connected places (local catalysis), distinct from remote sensing which operates through rate function global references without physical connection. Both connected channels and remote sensing provide concentration information: connected channels couple consumption with hierarchical broadcast through explicit topology for irreversible commitment, while remote sensing enables distance-independent regulation preserving local topology without preemption influence.

**Remote sensing (Rate functions** Φ**):** Implement mathematical dependencies where transitions reference places outside arc-connected locality. A transition *t* can sense *M*(*p*_remote_) without any arc $(p_{\text{remote}},t)\in F\cup F_{s}\cup F_{t}$ connec*t*ing them. This enables distance-independent regulation through kinetic equations $\Phi (t)=f(M(p_{1}),M(p_{2}),...,M(p_{\text{remote}}))$ where places *p*_remote_ may be topologically distant. Remote sensing operates through equation-implicit references without token transfer. Critically, the remote-sensed place *p*_remote_ need not belong to Ψ, need not carry any $F_{s}$ arc, and need not participate in the commitment path in any way. A Layer-3 execution sigma factor can sense a Layer-0 metabolite via Φ (feedback inhibition); a metabolic rate can depend on a downstream product (end-product inhibition); any place in *P* may appear in any rate function. Because Φ creates no $F_{s}$ arc, none of these dependencies appear in $G_{s}$ and none can create a preemption cycle. The acyclicity constraint governs only $G_{s}$ topology; Φ is completely unrestricted in its read set. Table 1 summarises the key differences between the two access modes.

**Table 1:** Connected vs. Remote Information Propagation.

Property	Connected ($F_{s}$)	Remote (Φ)
Mechanism	Physical arc	Mathematical dependency
Topology	Explicit in graph	Implicit in equations
Token transfer	Consumptive: $M^{\prime }=M-W_{s}$	Observational only
Locality	Direct connection	Any place
Vertical flow	Yes (layers ℓ>k)	No (local only)
Preemption	Hierarchical disabling	No hierarchical effect
Reversibility	Irreversible	Reversible
Example	GTP_pool $\overset{F_{s}\;}{\to }$ *T*_septation_	$\sigma_{H}$ gates *T*_septation_ via $\Phi (\sigma_{H}^{2})$

**Remark (**Φ **and** $F_{s}$ **serve structurally distinct roles):** Rate functions Φ are inherited unchanged from standard Bio-PN. This follows the two-layer architecture of stochastic Petri nets [13]: the *structural enabling* layer (Enabled predicate, topology-based) determines *whether* a transition may fire; the *kinetic propensity* layer (Φ) determines *how fast* it fires. Φ therefore does not appear in the Enabled predicate or the Firing rule by design—this is the standard separation in stochastic PN, not a deficiency. Φ governs simulation dynamics (propensity for τ-leaping, ODE rates). A natural question is whether Φ, being able to encode any function of any state variable, makes $F_{s}$ redundant. It does not: Φ operates over $G_{E}$—modelling continuous, reversible kinetics whose read dependencies may be cyclic—while $F_{s}$ operates over $G_{s}$—modelling discrete, irreversible commitment whose topology must be acyclic. The structural theorems (Acyclicity, Preemption, Basin Partition) are properties of $G_{s}$: they cannot be recovered from rate functions alone because Φ is not subject to the DAG constraint that makes them provable. In the limit—a net with all regulation in Φ and no arcs—the marking never changes from *M*_0_ and there is no basin boundary. The moment you add a signal flow arc you get an irreversible commitment boundary with threshold $M_{\text{commit}}=\theta +W_{s}$. Φ expresses what a transition *reads*; only $F_{s}$ arcs determine what it *destroys*, and it is destruction that makes commitment irreversible.

### 3.3. Hierarchical enablement

**Definition 4** (Hierarchical Enablement Predicate). *Transition t is hierarchically enabled at marking M if:*


$$\begin{array}{cc} Enabled(t,M) & \equiv NormalEnabled(t,M)\\ & \wedge TestEnabled(t,M)\\ & \wedge SignalEnabled(t,M)\\ & \wedge PreemptionCheck(t,M) \end{array}$$ (1)

where we define notation:

${\;}^{•}t=\{p\in P:(p,t)\in F\}$ (normal input places)
- $t^{•}=\{p\in P:(t,p)\in F\}$ (normal output places)
- ${\;}_{s}^{•}t=\{p_{s}\in \Psi :(p_{s},t)\in F_{s}\}$ (signal input places)
- $t_{s}^{•}=\{p_{s}\in \Psi :(t,p_{s})\in F_{s}\}$ (signal output places)
- ${\;}_{s}^{•}p=\{t\in T:(t,p)\in F_{s}\}$ (transitions producing place via signal arcs)
- $p_{s}^{•}=\{t\in T:(p,t)\in F_{s}\}$ (transitions consuming place via signal arcs)
- $\text{Test}(t)=\{p\in P:(p,t)\in F_{t}\}$ (test arc places)

and predicates:


$$\begin{array}{cc} \text{NormalEnabled}(t,M) & \equiv \forall p\in {\;}^{•}t:M(p)\geq W((p,t)) \end{array}$$ (2)

$$\begin{array}{cc} \text{TestEnabled}(t,M) & \equiv \forall p\in \text{Test}(t):M(p)>\tau_{t}((p,t)) \end{array}$$ (3)

$$\begin{array}{cc} \text{SignalEnabled}(t,M) & \equiv \forall p_{s}\in {\;}_{s}^{•}t:M(p_{s})\geq \theta (t)+W_{s}((p_{s},t)) \end{array}$$ (4)

$$\begin{array}{cc} \text{PreemptionCheck}(t,M) & \equiv \forall p_{s}\in {\;}_{s}^{•}t:\forall t^{\prime }\in {\;}_{s}^{•}p_{s}:\\ & \;(\text{NormalEnabled}(t^{\prime },M)\wedge \text{TestEnabled}(t^{\prime },M)\\ & \;\wedge \text{SignalEnabled}(t^{\prime },M)) \end{array}$$ (5)

The PreemptionCheck enforces hierarchical dependencies by requiring all transitions producing input signal places (via signal flow arcs) satisfy their local enablement conditions. This is a single-layer check: only immediate signal-producing predecessors are verified, not transitive ancestors. Hierarchical consistency propagates automatically because each layer’s PreemptionCheck verifies its direct predecessors, creating cascading verification back to Layer 0 through the acyclic structure.

### 3.4. Firing rule

**Definition 5 (Firing Function).** *Upon firing enabled transition t at marking M, the firing function Fire(t,M) produces new marking* $M^{\prime }$ *where:*


$$\begin{array}{c} M^{\prime }(p)=M(p)+\Delta_{normal}(p,t)+\Delta_{signal}(p,t) \end{array}$$ (6)

*with token changes:*


$$\begin{array}{c} \Delta_{normal}(p,t)=\left\{ \begin{array}{cc} -W((p,t))+W((t,p)) & if(p,t)\in F\;or\;(t,p)\in F\\ 0 & otherwise \end{array}\right. \end{array}$$ (7)

$$\begin{array}{c} \Delta_{\text{signal}}(p,t)=\left\{ \begin{array}{cc} -W_{s}((p,t))+W_{s}((t,p)) & if(p,t)\in F_{s}\;or\;(t,p)\in F_{s}\\ 0 & otherwise \end{array}\right. \end{array}$$ (8)

Test arcs do not affect marking: $(p,t)\in F_{t}$ implies $M^{\prime }(p)=M(p)$ (non-consuming observation).

**Remark (sensing threshold vs. kinetic weight):** The predicate TestEnabled uses the sensing threshold $\tau_{t}((p,t))\geq 0$, which is distinct from the kinetic weight $W_{t}((p,t))$ appearing in the rate function Φ(t). $\tau_{t}$ encodes the activation level of the local wired sensor—the minimum signal required to declare the transition enabled—while $W_{t}$ is a kinetic modulation parameter (e.g., $K_{m}$ in a Michaelis-Menten term). These quantities have different scaling laws: when initial conditions are rescaled by factor α, explicitly-set $\tau_{t}>0$ must scale by α to preserve enablement semantics, while $W_{t}$ (an intensive parameter embedded in Φ) is unchanged. The default $\tau_{t}=0$ implements a pure *presence check* (*M(p)* > 0), using the strict inequality in the TestEnabled predicate, which is scale-invariant and biologically appropriate when catalytic action requires only that the species be present.

**Dual arc case:** When p∈Ψ with both (p,t)∈F and $(p,t)\in F_{s}$, firing consumes tokens from both normal and signal weights: $M^{\prime }(p)=M(p)-W((p,t))-W_{s}((p,t))+W((t,p))+W_{s}((t,p))$.

Signal consumption depletes regulatory tokens, creating state space partitions: markings below commitment threshold cannot reach markings above after firing.

**Algorithm 1 Hierarchical Layer Assignment**

**Require:** Signal flow graph $G_{s}=(\Psi ,F_{s})$

**Ensure:** Layer function $\lambda :\Psi \to {\mathbb{N}}_{0}$ or FAIL if $G_{s}$ contains cycle

1: λ(p)←0 for all p∈Ψ

2: $\text{indeg}(p)\leftarrow |\{(p^{\prime },t)\in F_{s},(t,p)\in F_{s}\}|$ for all p∈Ψ     ▷ Count incoming signal paths

3: Q←{p∈Ψ:indeg(p)=0}               ▷ Source signal places

4: processed←0

5: **while** Q≠∅ **do**

6:  p←Q.dequeue()

7:  processed←processed+1

8:  **for** each $(p,t)\in F_{s}$ and $(t,p^{\prime })\in F_{s}$ **do**     ▷ Outgoing signal paths through *t*

9:   $\lambda (p^{\prime })\leftarrow \max(\lambda (p^{\prime }),\lambda (p)+1)$

10:  $\text{indeg}(p^{\prime })\leftarrow \text{indeg}(p^{\prime })-1$

11:   **if** $\text{indeg}(p^{\prime })=0$ **then**

12:     $Q.\text{enqueue}(p^{\prime })$

13:   **end if**

14:  **end for**

15: **end while**

16: **if** processed<|Ψ| **then**

17:  **return** FAIL               ▷ Cycle detected

18: **else**

19:  **return** λ

20: **end if**

### 3.5. Layer assignment algorithm

**Complexity:** $O(|\Psi |+|F_{s}|)$ via modified topological sort. Algorithm fails if $G_{s}$ contains cycles, providing constructive verification of acyclicity theorem.

### 3.6. Commitment Semantics

Commitment occurs when transition fires from basin boundary marking:

1. **Pre-commitment** (before firing): $M(p_{s})<M_{\text{commit}}(p_{s})$ — signal not yet at threshold, transition disabled, cell uncommitted
2. **Commitment boundary**: $M(p_{s})=M_{\text{commit}}(p_{s})$ — transition enabled; firing creates irreversibility
3. **Post-commitment** (after firing): $M^{\prime }(p_{s})=\theta (t)<M_{\text{commit}}(p_{s})$ — signal tokens consumed, residual marking prevents return; cell committed

The key insight: signal consumption creates one-way transition. Unlike normal Petri nets where reverse firing can restore markings, signal depletion establishes state space partitioning—committed cells cannot uncommit through any firing sequence. Once a cell commits to sporulation by firing $t_{commit}$ from basin boundary, consuming signal tokens, there is no alternative biochemical path back to the vegetative state through the same connected hierarchy. The commitment becomes final—the cell must follow the sporulation program forward. This captures biological reality: developmental commitment proceeds irreversibly through forward-only paths, even though metabolic feedback loops maintain homeostasis through remote sensing mechanisms.

**Fig 2:**
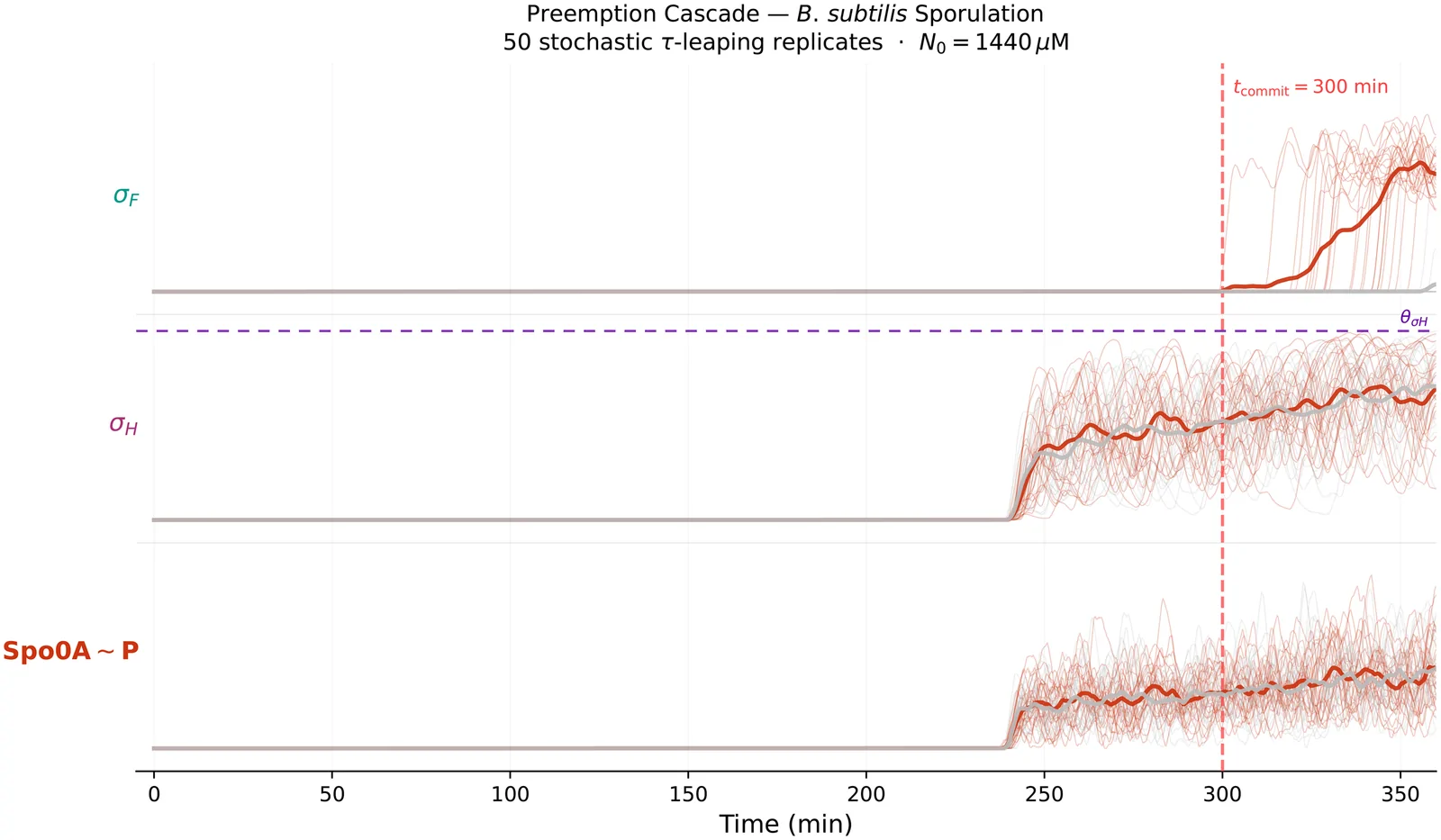
PreemptionCheck in action: stochastic commitment collapses to quasi-continuous execution. Individual trajectories of three key cascade species across 50 stochastic τ-leaping replicates of the *B. subtilis* sporulation model ($N_{0}=1440\;\mu$M, SinR =12μM, run_20260719_170034). Red: sporulating replicates (24/50); grey: vegetative replicates (26/50). Thick lines: fate-conditioned means. *Bottom* — Spo0A~P: stochastic phosphorelay; both fates share indistinguishable variance until $t_{commit}$. *Middle* — $\sigma_{H}$: commitment gate; violet dashed line marks $\theta_{\sigma H}=1.60\;\mu$M; only sporulating replicates cross it. *Top* — $\sigma_{F}$: execution programme; silent in vegetative replicates; activates post-commitment with markedly reduced variance, demonstrating the quasi-continuous character of execution once PreemptionCheck unlocks. The $\sigma_{H}$ effective threshold ($\theta_{\sigma H}=1.60\;\mu$M, identified via simulation) separates sporulating from vegetative replicates: once crossed, GTP tokens are consumed irreversibly via the signal-flow arc and all downstream layers fire in lock-step.

The three-phase commitment semantics are directly observable in these trajectories (Fig 2). Before t≈240min all 50 replicates are in the pre-commitment phase ($M(\sigma_{H})<\theta_{\sigma H}$) and all downstream layers are structurally disabled by PreemptionCheck regardless of Spo0A~P abundance. At $t_{commit}=300\;min$ the stochastic $\sigma_{H}$ ramp crosses $\theta_{\sigma H}$ in a subset of replicates: these enter the commitment boundary and fire $T_{septation}$, consuming GTP tokens via the signal-flow arc irreversibly. Post-commitment, all downstream layers ($\sigma_{F}$, $\sigma_{E}$, $\sigma_{G}$, $\sigma_{K}$, forespore assembly) fire in near-deterministic sequence — the red fan in the top strip is visibly tighter than the phosphorelay fan below, quantifying the noise suppression imposed by hierarchical gating. Replicates that never cross $\theta_{\sigma H}$ (grey) remain in the vegetative basin with $\sigma_{F}\equiv 0$ indefinitely: no alternative firing sequence can reach the sporulation attractor.

## 4. Signal hierarchical petri nets

We formalize signal hierarchical control as 13-tuple extension of classical Bio-PN, providing complete mathematical specification enabling computational implementation.

### 4.1. Formal definition

**Definition 6** (Signal Hierarchical Petri Net). *A Signal Hierarchical Petri Net is a 13-tuple:*


$$\begin{array}{c} \text{SPN}=(P,T,F,W,M_{0},\Phi ,C,F_{t},\Psi ,F_{s},W_{s},\lambda ,\theta ) \end{array}$$ (9)

*where:*

- *(P, T, F, W, M*_*0*_ *) is classical 5-tuple (places, transitions, arcs, weights, initial marking)*
- Φ:T→RateFunctions *assigns kinetic rate laws to transitions.* Φ *governs the simulation dynamics (propensity for* τ*-leaping, ODE rates). Following the standard two-layer architecture of stochastic Petri nets* [13], Φ *does not appear in the structural Enabled predicate: enabling is topological, propensity is kinetic.*
- C:P→Compartments *assigns each place to a spatial compartment (default: cytoplasm), used by the simulation engine for volume-based concentration conversion (*$c=N/(N_{A}\times V_{C})$*) and multi-compartment diffusion. C does not appear in the structural Enabled predicate or Firing rule: compartment assignment is a simulation-level parameter, not a topological one.*
- $F_{t}\subseteq P\times T$ *defines test arcs (catalysis, non-consuming); test arc enablement requires* $M(p)>\tau_{t}((p,t))$ *with default* $\tau_{t}=0$ *(presence check M(p) > 0) — the sensing threshold is uniformly 0 unless explicitly set*
- Ψ⊆P *defines signal places (information channels)*
- $F_{s}\subseteq (\Psi \times T)\cup (T\times \Psi )$ *defines signal flow arcs*
- $W_{s}:F_{s}\to {\mathbb{R}}^{+}$ *assigns signal arc weights*
- $\lambda :\Psi \to {\mathbb{N}}_{0}$ *assigns hierarchical layers*
- $\theta :T\to {\mathbb{R}}^{\geq 0}$ *assigns enablement thresholds*

### 4.2. Architectural principles

**Vertical-Horizontal Separation:** Normal arcs *F* model horizontal mass transfer (metabolic reactions), signal flow arcs $F_{s}$ model vertical information propagation (regulatory control). Signal places $p_{s}\in \Psi$ function as information hubs: metabolic transitions connect via normal arcs to modify $M(p_{s})$ at Layer 0 (horizontal participation), while regulatory transitions connect via signal flow arcs to consume signals and broadcast state changes to higher layers (vertical participation). This architectural separation mirrors biological organization where metabolic networks operate horizontally through spatially-distributed enzyme cascades, while regulatory hierarchies operate vertically with metabolic state gating developmental decisions through connected signal paths.

**Connected vs. Remote Information Transfer:** The formalism provides two complementary mechanisms. Connected channels (signal flow arcs $F_{s}$) implement topology-explicit wired propagation where physical arc connections enable hierarchical preemption—consumption at layer *k* triggers re-evaluation at all layers ℓ>k. Remote sensing (rate functions Φ) implements equation-implicit wireless observation where transitions reference concentrations of topologically distant places without physical connection—enabling long-range regulation through kinetic dependencies $\Phi (t)=f(M(p_{\text{remote}}))$ without hierarchical influence. Biologically, connected channels model committed resource allocation with hierarchical consequences ($\sigma_{H}$ depletion below $\theta_{\sigma H}=1.60\;\mu$M at commitment disables all downstream execution layers), while remote sensing models long-range regulatory effects without physical transport (Spo0A~P concentration influencing distant operon expression through equation dependencies).

**Dual Arc Semantics:** Signal places $p_{s}\in \Psi$ can simultaneously participate in both normal arcs (horizontal mass transfer) and signal flow arcs (vertical information propagation):


$$\begin{array}{cc} \text{Normal arc}: & \;(p_{s},t)\in F\text{ with weight }W((p_{s},t))\in {\mathbb{R}}^{+}\\ & \;\text{(stoichiometric consumption)} \end{array}$$ (10)

$$\begin{array}{cc} \text{Signal arc}: & \;(p_{s},t)\in F_{s}\text{ with weight }W_{s}((p_{s},t))\in {\mathbb{R}}^{+}\\ & \;\text{(regulatory quota)} \end{array}$$ (11)

When transition *t* fires with both arc types from place $p_{s}$, marking evolution is:


$$\begin{array}{c} M^{\prime }(p_{s})=M(p_{s})-W((p_{s},t))-W_{s}((p_{s},t))+W((t,p_{s}))+W_{s}((t,p_{s})) \end{array}$$ (12)

This enables the same metabolite (e.g., ATP) to participate in biochemical reactions (glycolysis) and regulatory decisions (commitment gating) without duplication.

**Design principle — selective wiretap on the physical layer:** Signal type assignment does not introduce new arcs, new places, or new tokens. It is a *selective annotation*: the modeler designates certain existing normal arcs as signal flow arcs, so that the same token-consuming event that performs biochemistry in $G_{E}$ simultaneously reports a commitment to the control hierarchy $G_{s}$. The cell does not maintain two separate copies of a molecule—one for metabolism, one for signaling. There is one token; the type annotation gives its consumption a second, hierarchical meaning. This is why the two-graph architecture is necessary: $G_{E}$ must be free to cycle (metabolic feedback, oscillations, reversible reactions), while $G_{s}$ must be acyclic (commitments are one-way). The same token stream feeds both graphs; the arc type determines which graph records the event.

### 4.3. Operational semantics

**Definition 7** (Marking Evolution). *State space* $ℳ={\mathbb{N}}_{0}^{\left|P\right|}$ *consists of all possible token distributions. Single-step reachability relation is:*


$$\begin{array}{c} M\overset{t\;}{\to }M^{\prime }⟺\;Enabled(t,M)\wedge M^{\prime }=Fire(t,M) \end{array}$$ (13)

*Multi-step reachability* $M\overset{*\;}{\to }M^{\prime }$ *is the reflexive transitive closure:* $M\overset{*\;}{\to }M^{\prime }$ *holds if there exists firing sequence* $t_{1},t_{2},...,t_{n}$ *with* $M\overset{t_{1}\;}{\to }M_{1}\overset{t_{2}\;}{\to }\cdots \overset{t_{n}\;}{\to }M^{\prime }$ *for some* n≥0.

**Definition 8** (Reachability Set). *The reachability set from initial marking M*_*0*_ *is:*


$$\begin{array}{c} ℛ(M_{0})=\{M\in ℳ:M_{0}\overset{*\;}{\to }M\} \end{array}$$ (14)

*Signal consumption creates reachability partition:* $ℛ(M_{0})=B_{below}\cup B_{boundary}\cup B_{above}$ *where basin boundaries form one-way barriers (Theorem: Irreversible Basin Partition).*

**Proposition 1** (Hierarchical Decomposition). *For signal hierarchical PN with k layers, reachability analysis decomposes into layer-wise subproblems. Analyzing Layer* ℓ *with fixed commitments from layers* 0,...,ℓ−1 *reduces complexity from* $O(\prod_{i=0}^{k}|M_{{Layer}_{i}}|)$ *(product of layer state spaces) to* $O(\sum_{i=0}^{k}|M_{{Layer}_{i}}|\cdot b_{i})$ *where* $b_{i}$ *is branching factor at layer i.*

Proof. General reachability in Petri nets is Ackermann-complete [19] (and hence not primitive-recursive), with state space that grows non-primitively-recursively in the number of places. For flat model with $\sum_{i}n_{i}$ places distributed across k layers, reachability graph has $O(\prod_{i}C^{n_{i}})$ states where C is token bound.

Hierarchical structure enables decomposition: (1) Analyze Layer 0 independently, computing commitment thresholds via $M_{\text{commit}}=\theta +W_{s}$ (*O*(1) per transition). (2) Fix Layer 0 commitments, reducing Layer 1 to subproblem with Layer 0 constraints—state space now $O(C^{n_{1}}\cdot b_{0})$ where b_0_ bounds Layer 0 commitment states. (3) Repeat for layers 2,...,k.

Total complexity: sum over layers instead of product. For B. subtilis model (40 places, 4 layers): flat analysis requires *O*(10^40^) states; hierarchical analysis requires $O(4\cdot 10^{10})$ states—a reduction of 30 orders of magnitude that enables practical computation. □

### 4.4. Comparison with classical Bio-PN

SHPN extends classical Bio-PN expressiveness through signal-type semantics, as summarised in Table 2.

**Table 2:** Expressiveness Comparison: Classical Bio-PN vs. Signal Hierarchical PN.

Capability	Classical	Signal Hier.
Mass transfer	Yes	Yes
Catalysis (non-consuming)	Yes	Yes
Regulatory consumption	No	Yes
Hierarchical layers	No	Yes
Threshold semantics	No	Yes
Basin boundaries	No	Yes
Commitment irreversibility	No	Yes
Unified metabolic-regulatory	No	Yes
Topology-driven thresholds	No	Yes

**Key distinction:** Test arcs enable transitions when $M(p)>\tau_{t}((p,t))$ without token consumption, maintaining $M^{\prime }(p)=M(p)$ after firing. Signal flow arcs require $M(p_{s})\geq \theta +W_{s}$ and consume tokens, producing $M^{\prime }(p_{s})=M(p_{s})-W_{s}$ after firing. This semantic difference between consumption and read-only behavior determines expressiveness: test arcs model catalysis such as enzyme-substrate binding, while signal flow arcs model commitment through decision quota depletion.

## 5. Unified metabolic-regulatory modeling

Signal hierarchical Petri nets enable integrated modeling where metabolic and regulatory networks share species through dual arc semantics. We demonstrate unified framework capability through multi-layer architecture.

### 5.1. Signal place designation

The formalism’s power derives from modeler choice: any metabolite can be designated as signal place $p_{s}\in \Psi$ when biological evidence supports regulatory gating role. This designation determines dual participation through horizontal mass transfer and vertical information broadcast.

**Horizontal mass transfer.** Signal places are normal places participating in biochemical reactions via normal arcs *F* at Layer 0. For example, ATP in glycolysis: $(t_{\text{glycolysis}},\text{ATP})\in F$ with *W* = 2 (stoichiometric production). NADH in oxidative phosphorylation: $(\text{NADH},t_{\text{complex-I}})\in F$ with *W* = 1 (consumption).

**Vertical information broadcast.** The same places, when we assign type “signal,” broadcast availability to higher regulatory layers via signal flow arcs $F_{s}$. For example, GTP_pool gating septation: $(\text{GTP\_pool},\;T_{\text{septation}})\in F_{s}$ with $W_{s}>0$ (structural commitment arc consumed on firing). Spo0A~P broadcasting phosphorelay output: $(\text{Spo0A}\sim \text{P},\;t_{\sigma H})\in F_{s}$ (integration-to-commitment gating). Note that $\sigma_{H}$ gates *T*_septation_ kinetically via rate function $\Phi (T_{\text{septation}})\propto [\sigma_{H}{]}^{2}$ (remote sensing), not via a signal flow arc—the commitment separatrix $\theta_{\sigma H}=1.60\;\mu$M is a kinetic threshold identified by simulation, not a structural $M_{\text{commit}}=\theta +W_{s}$ value.

This type assignment creates an interface from Layer 0 to higher layers: signal places and signal flow arcs are ordinary places and arcs to which we assign signal semantics, enabling the same places to participate in horizontal mass flow (metabolic transformations) and vertical information broadcast (regulatory propagation). Mass flows horizontally through stoichiometric reactions; information about mass availability broadcasts spatially (vertically) to the cellular regulatory environment. No artificial separation into distinct species—unified place, differentiated semantics through arc type assignment.

Biological case selection: For *B. subtilis* sporulation, we designate $\sigma_{H}$ as the commitment-gate signal place and Spo0A~P as the phosphorelay integration signal [14]. ATP and GTP remain metabolic participants (via normal arcs) but are not the separatrix species: the commitment threshold is the $\sigma_{H}$ separatrix ($\theta_{\sigma H}=1.60\;\mu$M), not an energy quota. Other systems designate different signals: calcium (Ca^2+^) for neurotransmitter release, cyclic AMP (cAMP) for bacterial chemotaxis, GTP for protein synthesis quality control, NADH for metabolic oscillations. The formalism generalizes across signal types—designation derives from biological function, not molecular identity.

### 5.2. Four-layer architecture with multi-level signal assignment

Signal places can be designated at *any* hierarchical layer, not restricted to Layer 0. This design choice significantly enhances formalism expressiveness by enabling multi-stage commitment with cascading irreversibility. For the *B. subtilis* case, signal places are designated across all four layers as determined by the signal flow arc topology of the v9 model:

**Layer 0 (Starvation Sensing):** KinA_kinase_ designated as signal ($\lambda ({\text{KinA}}_{\text{kinase}})=0$). Rate $\propto {\text{KinA}}_{\text{kinase}}\cdot 1/(1+{\text{Nutrients}}^{2})$: active only under starvation. Signal flow arcs connect KinA_kinase_ to the phosphorelay entry, creating the first commitment barrier: absence of nutrient stress structurally disables all downstream layers regardless of phosphorylation state.

**Layer 1 (Phosphorelay Integration):** Spo0A~P designated as signal (λ(Spo0A~P)=1). Signal flow arcs carry the phosphoryl group through the four-protein relay (KinA_kinase_ → KinA_P_ → Spo0F_P_ → Spo0A~P), each step stochastic. Spo0A~P broadcasts to Layer 2, creating the second commitment barrier: insufficient phosphorelay output prevents commitment-gate activation.

**Layer 2 (Commitment Gate):** $\sigma_{H}$ designated as signal ($\lambda (\sigma_{H})=2$). Produced by a Hill-4 positive-feedback switch on Spo0A~P ($T_{\sigma H}$: $\Phi \propto \text{Spo0A}\sim {\text{P}}^{4}/(50+\text{Spo0A}\sim {\text{P}}^{4})$); gates *T*_septation_ kinetically via remote sensing ($\Phi \propto \sigma_{H}^{2}$). The $\sigma_{H}$ effective threshold $\theta_{\sigma H}=1.60\;\mu$M is identified via simulation (Sweep A): only replicates crossing it fire *T*_septation_, which consumes GTP tokens via the structural signal-flow arc, unlocking Layer 3.

**Layer 3 (Execution):** $\sigma^{F}$, $\sigma^{E}$, $\sigma^{G}$, $\sigma^{K}$ designated as signals ($\lambda (\sigma^{F})=3$). Continuous sigma factor cascade activated post-septation. Signal flow arcs from Layer 2 unlock transcription; once active, execution proceeds with markedly reduced variance (quasi-continuous regime, visible in Fig 2): sigma factor depletion can no longer reverse the committed phenotype.

This multi-level signal architecture enables *cascading irreversibility*: each layer establishes its own basin boundaries through signal consumption. A cell can be starvation-committed (Layer 0 active) yet phosphorelay-uncommitted (Layer 1 below threshold), creating intermediate commitment states impossible if signals were restricted to a single layer. The formalism thus captures multi-stage biological decision-making with progressive commitment refinement.

### 5.3. Information flow topology

Signal flow graph $G_{s}$ encodes regulatory architecture through connected channels created by type assignment. Signal places function as hierarchical information hubs at each layer, not restricted to Layer 0. For the *B. subtilis* case, we designate $\Psi =\{{\text{KinA}}_{\text{kinase}},\text{Spo0A}\sim \text{P},\sigma_{H},\sigma^{F}\}$ spanning all four layers. Layer assignment $\lambda ({\text{KinA}}_{\text{kinase}})=0$, λ(Spo0A~P)=1, $\lambda (\sigma_{H})=2$, $\lambda (\sigma^{F})=3$ creates the multi-level signal architecture.

Connected paths define hierarchical preemption cascades. For example:

- $\left({\text{KinA}}_{\text{kinase}},\;T_{\text{KinA}},\;{\text{KinA}}_{P}\right)$: Layer 0 starvation gating (signal flow arc)
- $\left({\text{KinA}}_{P}\to {\text{Spo0F}}_{P}\to \text{Spo0A}\sim \text{P}\right)$: Layer 0→1 phosphorelay broadcast (signal flow chain)
- $\left(\text{Spo0A}\sim {\text{P}}^{4}\overset{\Phi \;}{\to }\sigma_{H}\right)$: Layer 1→2 Hill-switch commitment gate (rate function)
- $\left(\sigma_{H}^{2}\overset{\Phi \;}{\to }T_{\text{septation}}\to \sigma^{F}\right)$: Layer 2→3 execution unlock (rate function remote sensing)

Each signal place at layer *k* can be produced/consumed via normal arcs (horizontal mass transfer within layer) and broadcast via signal flow arcs to layer *k* + 1 (vertical information propagation across layers). This architecture enables *cascading commitment*: depletion at any layer preempts all higher layers through hierarchical preemption, while each layer maintains its own basin boundaries.

Acyclicity enforced: no connected path $\sigma^{F}⇝{\text{KinA}}_{\text{kinase}}⇝\sigma^{F}$ exists through signal flow arcs—execution sigma factors cannot re-activate the starvation sensor through hierarchical commitment channels. Remote sensing through rate functions Φ enables equation-implicit feedback without creating preemption cycles: $\sigma^{F}$ transcription can sense any place—including upstream metabolites, downstream products, or places with no $F_{s}$ arc at all—via $\Phi (\sigma^{F})=f(M(p_{\text{remote}}))$ without physical arc connection. The remote site is not required to be in the commitment path, not required to be a member of Ψ, and not required to have any $F_{s}$ arc. Feedback therefore remains fully unrestricted (metabolic oscillations, product inhibition, allosteric regulation) while the irreversible commitment structure stays acyclic. The formalism thus captures both hierarchical stratification (acyclic $G_{s}$) and full feedback biology (cyclic remote dependencies in Φ) independently.

### 5.4. Threshold computation

The formalism enables topology-driven threshold computation. For any designated signal $p_{s}\in \Psi$ controlling transition *t*, the commitment marking follows:


$$\begin{array}{c} M_{\text{commit}}(p_{s})=\theta (t)+W_{s}((p_{s},t)) \end{array}$$ (15)

This formula expresses commitment finality: firing from *M*_commit_ consumes decision quota $W_{s}$, leaving residual $M^{\prime }=\theta$ exactly—the minimal marking maintaining enablement. Below *M*_commit_, firing disabled. Above, firing possible but optional. At boundary, firing creates irreversibility through basin partitioning.

The formula generalizes across signal types: calcium-triggered neurotransmitter release designates Ca^2+^, cAMP-triggered chemotaxis designates cAMP, protein synthesis quality control designates GTP. Threshold computation depends on topology and biological designation, not molecular identity. The *B. subtilis* sporulation case demonstrates this principle with $\sigma_{H}$ as the designated commitment-gate signal ($\theta_{\sigma H}=1.60\;\mu$M) and Spo0A~P as the phosphorelay integration signal.

## 6. Validation: Mathematical proofs and biological case study

We validate signal hierarchical Petri nets through dual methodology: mathematical proofs establishing correctness, biological case study demonstrating predictive capability.

### 6.1. Mathematical validation

#### 6.1.1. Theorem: Acyclicity enforcement.

Signal hierarchical Petri nets algorithmically enforce acyclicity through layer assignment failure detection.

**Proposition 2** (Algorithm Correctness). *Layer assignment algorithm (Algorithm 1) succeeds if and only if* $G_{s}$ *is acyclic. When successful, the produced* λ *satisfies layer function requirements.*

*Proof.* (If acyclic ⇒ algorithm succeeds): Assume $G_{s}$ acyclic. Every DAG has at least one node with indegree 0 (no predecessors). Initially, *Q* contains all such nodes, so Q≠∅. When we process place *p* and remove its outgoing edges, we potentially reduce indegrees of successors to 0, adding them to *Q*. Since graph is finite and acyclic, this process terminates with all |Ψ| places processed.

For layer function validity, consider any signal flow path $p_{i}\overset{t\;}{\to }p_{j}$ where $(p_{i},t)\in F_{s}$ and $(t,p_{j})\in F_{s}$. Algorithm processes $p_{i}$ before $p_{j}$ (topological order). When processing $p_{i}$, line 13 sets $\lambda (p_{j})\leftarrow \max(\lambda (p_{j}),\lambda (p_{i})+1)$. Since $\lambda (p_{i})\geq 0$, this ensures $\lambda (p_{j})\geq \lambda (p_{i})+1>\lambda (p_{i})$. Hence $\lambda (p_{i})<\lambda (p_{j})$ as required.

(If algorithm succeeds ⇒ acyclic): Suppose algorithm succeeds with processed =|Ψ|. If cycle existed, say $p_{1}\overset{t_{1}\;}{\to }p_{2}\overset{t_{2}\;}{\to }\cdots \overset{t_{n}\;}{\to }p_{1}$, then all places in cycle have indeg ≥1 throughout execution (removing one incoming edge still leaves others from cycle). None reach indeg = 0, so none enter *Q*, thus processed <|Ψ|—contradiction. Therefore $G_{s}$ must be acyclic. □

This provides computational verification: attempt layer assignment; if success, $G_{s}$ valid and hierarchy established; if failure, circular control dependencies detected. Biological models automatically checked for structural validity.

#### 6.1.2. Theorem: Preemption correctness.

Hierarchical enablement predicate correctly implements preemption semantics.

**Proposition** 3 (Hierarchical Consistency). *If Enabled(t, M) = true under hierarchical predicate, then all lower-layer signal place producers satisfy their enablement thresholds.*

*Proof.* We prove by strong induction on layer depth $\ell =\lambda_{\max}(t)$ where $\lambda_{\max}(t)=\max\{\lambda (p_{s}):p_{s}\in {\;}_{s}^{•}t\}$ is the maximum layer of signal inputs to *t*.

**Base case** (ℓ=0): Transition *t* has no signal inputs from higher layers, only Layer 0 metabolic places. PreemptionCheck(*t*,*M*) vacuously true (no lower-layer predecessors to check). SignalEnabled(*t*,*M*) requires only Layer 0 *t*hresholds, which are satisfied by definition of Enabled(*t*,*M*).

**Inductive step**: Assume for all transitions $t^{\prime }$ with $\lambda_{\max}(t^{\prime })<\ell$, if Enabled$\left(t^{\prime },M\right)$ then all their lower-layer predecessors satisfy thresholds. Consider transition *t* with $\lambda_{\max}(t)=\ell >0$.

By definition of Enabled(*t*,*M*), we have PreemptionCheck(*t*,*M*) = true, which requires:


$$\begin{array}{cc} & \forall p_{s}\in {\;}_{s}^{•}t:\forall t^{\prime }\in {\;}^{•}p_{s}:\\ & \;(\text{NormalEnabled}(t^{\prime },M)\wedge \text{TestEnabled}(t^{\prime },M)\wedge \text{SignalEnabled}(t^{\prime },M)) \end{array}$$

For each signal input $p_{s}\in {\;}_{s}^{•}t$ with $\lambda (p_{s})\leq \ell$, all producing transitions $t^{\prime }\in {\;}_{s}^{•}p_{s}$ satisfy their local enablement conditions. Since $\lambda (p_{s})<\ell$, these transitions have $\lambda_{\max}(t^{\prime })\leq \lambda (p_{s})<\ell$. By inductive hypothesis applied to each $t^{\prime }$, all their direct signal predecessors satisfy thresholds.

Hierarchical consistency propagates through the acyclic structure: verifying immediate predecessors at each layer creates cascading verification along signal flow paths $p_{1}\overset{t_{1}\;}{\to }p_{2}\overset{t_{2}\;}{\to }\cdots \overset{t_{k}\;}{\to }p_{s}$. Each transition $t_{i}$’s PreemptionCheck verifies $t_{i-1}$, creating chain of verification back to Layer 0 sources without requiring explicit transitive checking. □

This guarantees ATP depletion cascades upward: if Layer 0 fails threshold, all dependent layers 1,2,...,ℓ disabled regardless of local token abundances.

#### 6.1.3. Corollary: Threshold predictability.

Basin boundaries are structural properties computable without simulation.

**Proposition 4** (Computational Threshold Prediction). *For transition t with signal input* $(p_{s},t)\in F_{s}$*, commitment threshold* $M_{\text{commit}}(p_{s})=\theta (t)+W_{s}((p_{s},t))$ *is computable in O(1) time from formalism specification without state space exploration.*

*Proof.* By Definition (Basin Boundary), commitment marking is defined as $M_{\text{commit}}(p_{s})=\theta (t)+W_{s}((p_{s},t))$. Both components are formalism parameters:

- θ(t) is specified via threshold function $\theta :T\to {\mathbb{R}}^{\geq 0}$ (part of 13-tuple definition)
- $W_{s}((p_{s},t))$ is specified via signal weight function $W_{s}:F_{s}\to {\mathbb{R}}^{+}$ (part of 13-tuple definition)

Computation procedure:

1. Identify signal input arc: $(p_{s},t)\in F_{s}$ (graph traversal, $O(|F_{s}|)$ worst case)
2. Retrieve threshold: $\theta_{\text{val}}=\theta (t)$ (function evaluation, *O*(1))
3. Retrieve weight: $w_{\text{val}}=W_{s}((p_{s},t))$ (function evaluation, *O*(1))
4. Compute commitment: $M_{\text{commit}}(p_{s})=\theta_{\text{val}}+w_{\text{val}}$ (addition, *O*(1))

No simulation, no reachability analysis, no numerical integration—direct arithmetic from model specification. This contrasts with the Ackermann-complete reachability analysis [19] required for general threshold determination in unstructured Petri nets. □

This establishes topology-driven predictability: the threshold is *structurally localized* — once the signal topology is fixed, it is determined by at most two measurable quantities per signal arc (θ and $W_{s}$), extracted from biological literature (BRENDA database, biochemistry textbooks). The claim is not that the threshold is parameter-free, but that it does not require fitting a global nonlinear model: extract parameters from biological literature, instantiate formalism functions θ and $W_{s}$, compute threshold via closed-form formula, obtain quantitative prediction without simulation or parameter optimization. For the *B. subtilis* sporulation model this yields the $\sigma_{H}$ effective commitment threshold, identified via simulation in Proposition 5 in Section 6.

**Remark (Kinetic constraint tuple** Γ **and effective basin floor** $\theta_{eff}$**):** Each signal flow arc carries a kinetic constraint tuple Γ=(K,n,ε) with three components drawn from published enzyme kinetics: K≥0 is the Michaelis constant (same units as marking, e.g., μM), *n* > 0 is the Hill coefficient (cooperativity exponent), and ε∈[0,1) is the suppression floor fraction. The effective basin floor is computed analytically from Γ:


$$\begin{array}{c} \theta_{eff}=K\cdot {\left(\frac{\varepsilon }{1-\varepsilon }\right)}^{1/n} \end{array}$$ (16)

When ε=0 (default), $\theta_{eff}=0$ and enablement reduces to $M(p_{s})\geq W_{s}$. The threshold component θ(t) of the 13-tuple evaluates to $\theta_{eff}$ for each signal input arc $\left(p_{s},t\right)$.

The claim that $M_{commit}=\theta_{eff}+W_{s}$ is “not fitted” operates at two distinct levels. *(1) Cross-domain grounding:* Γ=(K,n,ε) is sourced from molecular kinetics data (BRENDA, published Hill-equation fits) — a different experimental domain from the sporulation fraction being predicted. These parameters describe enzyme–substrate binding behaviour; they were not adjusted to match any cellular-level observation. *(2) Analytical derivation:* $\theta_{eff}$ and $M_{commit}$ are computed once by formula, with no tuning step targeting the Fujita fractions. The validation claim is therefore that molecular-scale constants, sourced independently, predict a cell-population-scale commitment threshold without any intermediate fitting targeting the predicted outcome.

### 6.2 Biological validation: *Bacillus subtilis* sporulation

#### 6.2.1 Experimental context.

*B. subtilis* enters sporulation—an irreversible developmental program producing dormant endospores—when nutrient depletion signals starvation. Fujita and Losick [14] demonstrated that sporulation outcome depends critically on the *rate and timing* of Spo0A~P accumulation: abrupt induction of constitutively active Spo0A* yielded only ≈5% sporulation efficiency (Fig 2C/D), whereas the identical Spo0A~P level reached gradually via the natural KinA phosphorelay yielded ≈52% (Fig 2A/B). Crucially, $\sigma_{H}$ is a necessary condition: $\sigma_{H}$-null mutants fail to sporulate even with Spo0A* induction (Fig. 4; 0% forespore formation). The mechanistic basis for this timing dependence is the central explanatory challenge addressed by this case study.

#### 6.2.2. Model construction.

**Fig 3:**
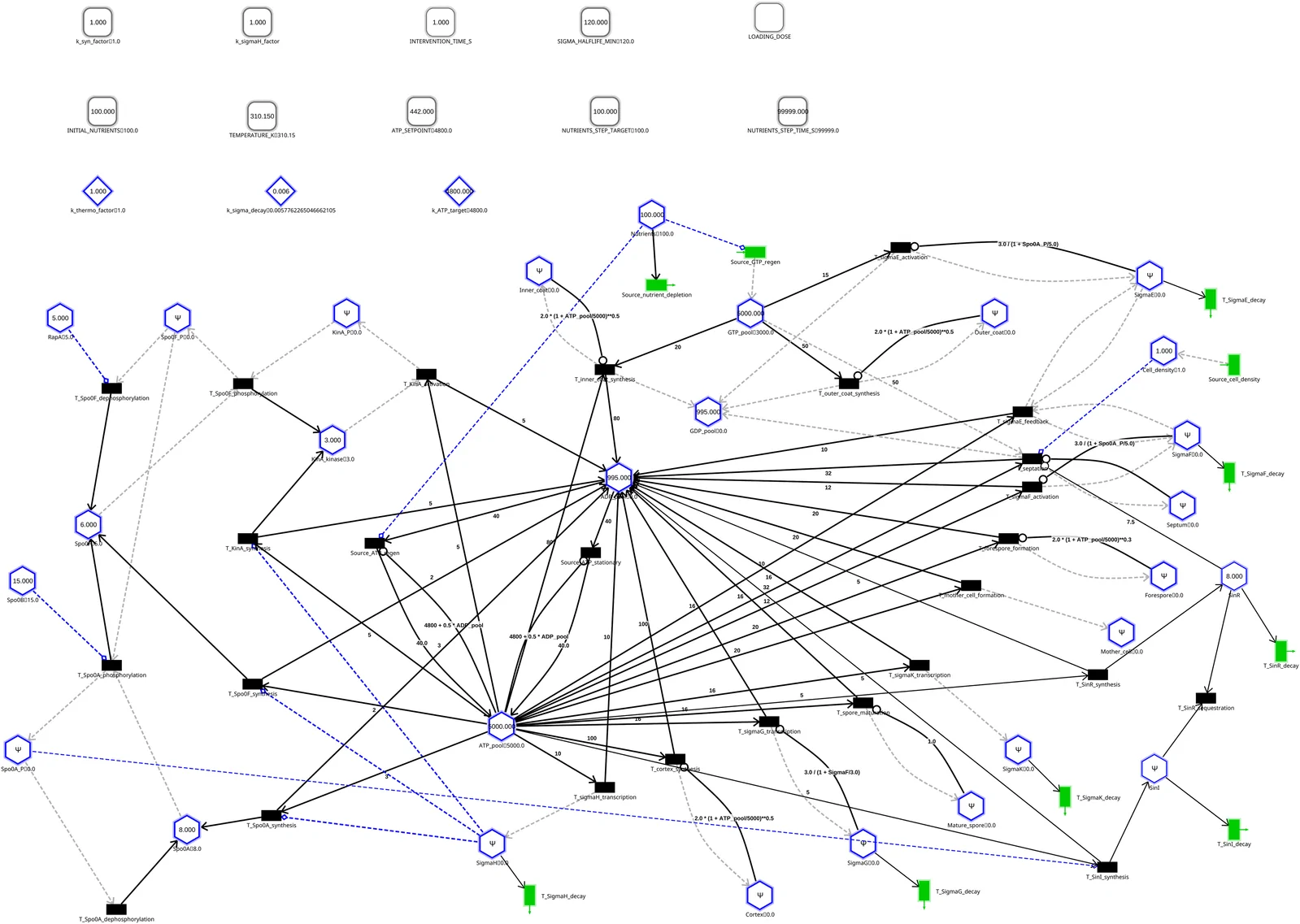
Signal Hierarchical Petri Net: *B. subtilis* Sporulation (v9). Full topology of the *B. subtilis* sporulation Signal Hierarchical Petri Net. Circles (•) are biological places; hexagons (Ψ) are signal places; diamonds (◊) are spatial-signal places; rounded squares (◻) are parameter places. Black solid lines: normal arcs (stoichiometric consumption); light-grey dashed lines: signal-flow arcs; blue dashed lines: test arcs (non-consuming, presence check only); green rectangles: source/sink transitions. The four signal layers are: Layer 0 (KinA_kinase_, starvation sensing), Layer 1 (Spo0A~P, phosphorelay integration), Layer 2 ($\sigma_{H}$, commitment gate, separatrix $\theta_{\sigma H}=1.60\;\mu$M), Layer 3 ($\sigma_{F}$, $\sigma_{E}$, $\sigma_{G}$, $\sigma_{K}$, execution programme). Parameter places (top row) encode the experimental protocol (nutrient level, temperature, loading dose) and are read by events only—they carry no arcs and do not appear in any rate function Φ.

The model instantiates the four-layer signal hierarchical architecture (Section 5.2). Model statistics: 40 places, 36 transitions, 114 arcs, 4 signal layers (Fig 3).

#### 6.2.3. Parameter sources.

Rate constants and kinetic parameters from independent biological sources; the sporulation efficiency ratio (48–52% vs. ≈5%) was not a fitting target:

- Glycolytic rate constants: BRENDA database [20]
- Phosphorelay kinetics: Biochemistry textbooks [21]
- Transcription/translation rates: *B. subtilis* genome annotations [22]
- Γ=(K,n,ε) for signal arcs: BRENDA database and published Hill-equation fits; not adjusted to match Fujita fractions
- $\sigma_{H}$ effective threshold $\theta_{\sigma H}\approx 1.60\;\mu$M: identified empirically via Sweep A bifurcation (run_20260611_231304); this is a kinetic threshold from $\Phi (T_{\text{septation}})\propto [\sigma_{H}{]}^{2}$, not a structural $M_{\text{commit}}=\theta +W_{s}$ computation
- SinR/SinI gate: T32 $K_{m}\approx 52.9\;\mu$M calibrated to match the stochastic bimodal gate dynamics (log-log interpolation across Sweep C, run_20260719_170034); calibration target was gate shape, not the 52% fraction

#### 6.2.4. Threshold prediction.

**Proposition 5** ($\sigma_{H}$ Separatrix and Sporulation Efficiency). *For the B. subtilis sporulation model,* $T_{septation}$ *is effectively enabled if and only if* $[\sigma_{H}]\geq \theta_{\sigma H}\approx 1.60\;\mu$*M, identifying a positive-feedback separatrix that couples commitment timing to the* $\sigma_{H}$ *accumulation ramp.*

*Proof.* $T_{septation}$ rate $=0.005\cdot [\sigma_{H}{]}^{2}\cdot [\text{Cell\_density}]\cdot \frac{\left[\text{ATP}\right]}{200+[\text{ATP}]}\cdot \frac{\left[\text{GTP}\right]}{100+[\text{GTP}]}$. The SinR inhibitor arc (threshold 7.5μM) structurally blocks $T_{septation}$ until the SinR/SinI gate opens stochastically (Sweep C mechanism). The $[\sigma_{H}{]}^{2}$ factor ensures the rate is negligible below $\theta_{\sigma H}$: at $[\sigma_{H}]=0.5\;\mu$M, rate ∝0.25; at $[\sigma_{H}]=1.60\;\mu$M, rate ∝2.56 (10× increase). Sweep A (run_20260611_231304) identifies this critical value directly from the k_syn_factor bifurcation: replicates below $\theta_{\sigma H}$ produce zero Septum; replicates above produce Septum = 1.00 at ≈330min post-starvation.□

#### 6.2.5. Sweep C: Fujita Perturbation Protocol (run_20260719_170034).

Sweep C directly implements the Fujita and Losick [14] experimental design across 25 conditions and 50 stochastic replicates each:

- **Natural route** ($N_{0}=1440\;\mu$M, LOADING_DOSE = 0): nutrient depletion at t≈4h; Spo0A~P accumulates gradually; $\left[\sigma_{H}\right]$ crosses $\theta_{\sigma H}$ at t≈5.5h; SinR/SinI gate opens stochastically. Result: 48% sporulation (24/50 replicates at SinR =12μM; range 44–55% across SinR levels, mean ≈52%). *Matches Fujita* Fig 2A*/B (*≈52%*)*.
- **Abrupt pulse** ($N_{0}=2160\;\mu$M, LOADING_DOSE =27μM at *t* = 1 s): nutrients never deplete; abrupt Spo0A~P boost cleared in ≈7s by nutrient-boos*t*ed Spo0A phosphatase (×10 rate at $N_{0}=2160\;\mu$M) before $\left[\sigma_{H}\right]$ builds; $\left[\sigma_{H}\right]$ never crosses $\theta_{\sigma H}$; minimal SinI produced. Result: ≈5% sporulation (0–6% depending on SinR level). *Matches Fujita* Fig 2C*/D (*≈5%*)*.

**Experimental comparison:**

- Fujita natural route ≈52%; model: 48% at SinR =12μM, ≈52% averaged across SinR levels (< 4% difference from Fujita reference).
- Fujita abrupt route ≈5%; model: 0--6% (*N*_0_ = 2160, dose = 27, SinR ≥4μM).
- No arc parameter was optimized to match these sporulation fractions. Γ=(K,n,ε) values for all signal arcs derive from published biological measurements (BRENDA, Voet & Voet [21]). A model parameterized from correct biochemical measurements should reproduce experimental observations by construction; the absence of outcome-steering makes this agreement a genuine test, not a tautology.

The PreemptionCheck mechanism underlying this asymmetry is made directly visible in individual replicate trajectories (see Fig 2, § 3.6): the stochastic $\sigma_{H}$ ramp sorts replicates into sporulating (red) and vegetative (grey) fates at the $\theta_{\sigma H}$ separatrix, after which the execution programme fires with markedly reduced inter-replicate variance.

#### 6.2.6. Basin of attraction analysis.

**Fig 4:**
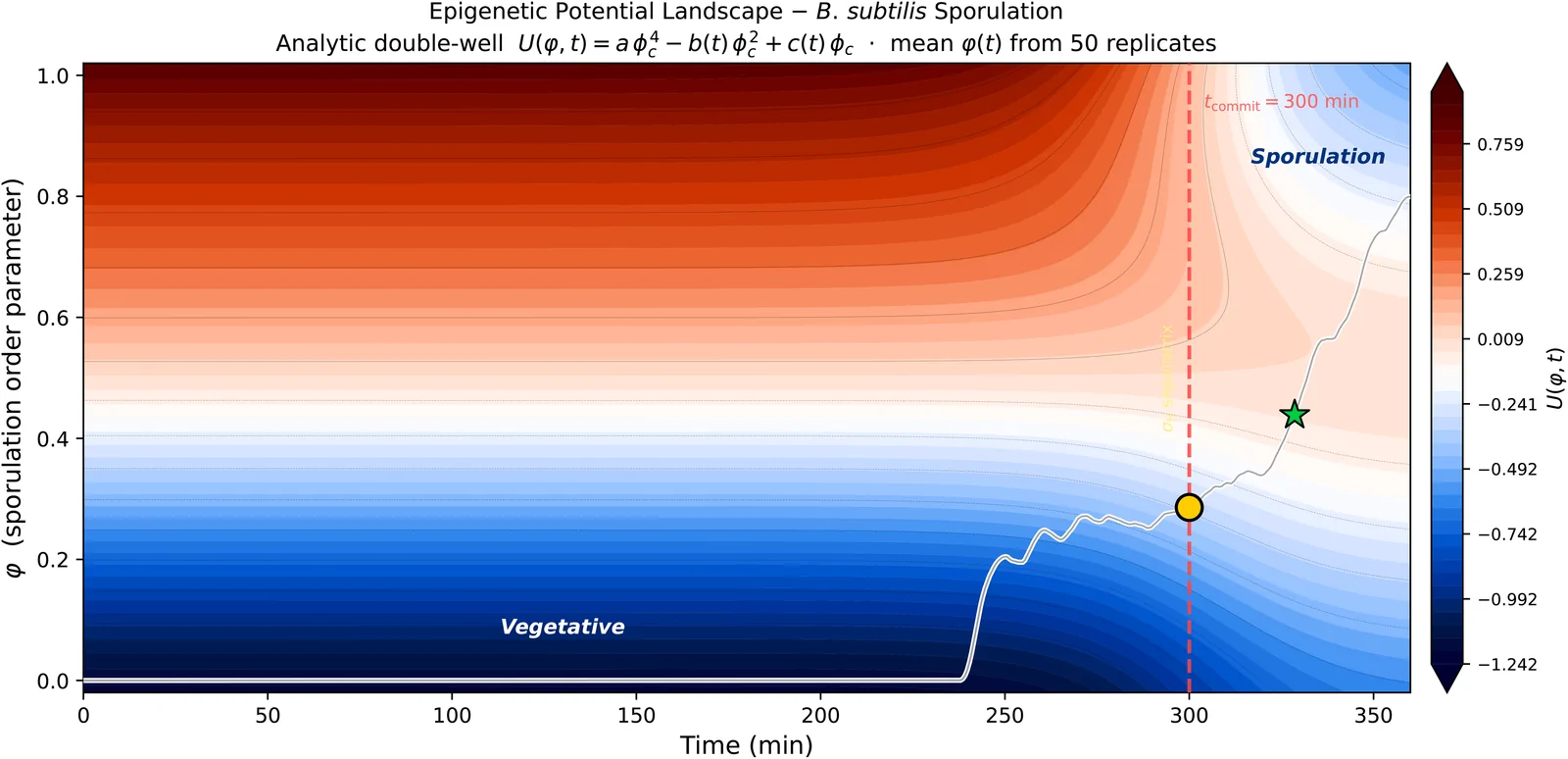
Waddington epigenetic potential landscape of *B. subtilis* sporulation commitment. Analytic quasi-potential $U(\varphi ,t)=a\;\varphi_{c}^{4}-b(t)\;\varphi_{c}^{2}+c(t)\;\varphi_{c}$ ($\varphi_{c}=\varphi -0.5$) with time-varying coefficients calibrated to the commitment clock ($t_{commit}=300\;min$, median $\sigma_{H}$ crossing at 1.60μM). Dark blue = low-energy attractor basins; dark red = energy barrier (Waddington ridge). White line: mean sporulation order parameter φ(t) averaged over 50 stochastic τ-leaping replicates (run_20260719_170034, $N_{0}=1440\;\mu$M, 6-h horizon). Yellow circle (•): commitment point at $t_{commit}$; green star (⋆): first appearance of mature spores (t≈329min). Red dashed line: $\sigma_{H}$ separatrix. The landscape reproduces the bet-hedging outcome reported by Fujita & Losick [14]: ≈48% sporulation under nutrient-rich conditions ($N_{0}=1440\;\mu$M, SinR =12μM, no drug pulse).

The landscape (Fig 4) reveals three dynamical regimes along the time axis. Before $t_{commit}\approx 300\;min$ the landscape is a single vegetative well (φ≈0): all 50 replicates are trapped at low order parameter regardless of stochastic fluctuations, because $[\sigma_{H}]<\theta_{\sigma H}$ and $T_{septation}$ remains blocked. Around $t_{commit}$ the barrier lifts (coefficients *b*(*t*) and *c*(*t*) transition via tanh), the ridge emerges, and the mean trajectory φ(t) begins to rise. After t≈329min a sporulation attractor forms in the upper region (φ→1), separating from the persistent vegeta*t*ive well a*t* φ≈0. The near-symmetric double-well at late times encodes the ≈48% sporulation fraction: neither basin is strongly favoured, reflecting the bet-hedging character of the commitment decision.

**Corollary 5** (Basin Boundary Characterization — *B. subtilis*). *For the B. subtilis sporulation model, the reachability space* $ℛ(M_{0})$ *partitions into two regions. Using the notation of Theorem (Irreversible Basin Partition) with the SinR/*$\sigma_{H}$ *commitment gate:*


$$\begin{array}{cc} B_{below} & =\{M\in ℛ(M_{0}):M(GTP\_pool)<M_{commit}(\text{GTP\_pool})\}\\ B_{boundary} & =\{M\in ℛ(M_{0}):M(GTP\_pool)=M_{commit}(GTP\_pool)\}\\ B_{above} & =\{M\in ℛ(M_{0}):M(GTP\_pool)>M_{commit}(GTP\_pool)\wedge T_{septation}\;has\;fired\} \end{array}$$

*where GTP_pool is the signal-flow arc input to T*_*septation*_ *and* $M_{commit}(GTP\_pool)=\theta (T_{septation})+W_{s}((GTP\_pool,T_{septation}))$*. The SinR inhibitor arc (threshold* 7.5μ*M) and the* $[\sigma_{H}{]}^{2}$ *kinetic factor constitute the complementary structural and kinetic commitment barriers. No firing sequence from B*_*below*_ *reaches B*_*above*_ *after T*_*septation*_ *fires.*

*Proof.* Direct application of Theorem (Irreversible Basin Partition) with GTP_pool as the signal-flow arc input to *T*_septation_. When the SinR/SinI gate opens and $\left[\sigma_{H}\right]$ is above the effective kinetic threshold, *T*_septation_ fires from *B*_boundary_, consuming GTP tokens via the signal-flow arc and producing Septum via the curved signal-flow arc. The resulting Septum inhibitor arc ($\text{Septum}\to T_{\text{septation}}$, curved inhibitor) then blocks re-firing, making the partition absorbing. With no $F_{s}$ arc regenerating GTP_pool after consumption, *B*_below_ is absorbing per the theorem. □

#### 6.2.7. Sensitivity analysis.

**Proposition 6** (Threshold Prediction Robustness). *For parameter perturbations* δ∈[−20%,+20%] *applied to model components (phosphorelay kinetics, SinR/SinI rates,* $\sigma_{H}$ *feedback strength, nutrient depletion rate), sporulation fraction (Sweep C, N*_*0*_ *= 1440, dose = 0) remains within [42%, 62%], and abrupt-pulse fraction (N*_*0*_ *= 2160, dose = 27) remains within [0%, 10%], preserving the qualitative asymmetry.*

*Verification (empirical, not proof):* This robustness bound was verified by varying each parameter class by ±20% in sweep simulations.

- **Phosphorelay kinetics (**±20%**):** natural sporulation 42--62%; abrupt < 10%.
- **SinR/SinI gate (**±20%**):** natural 40--64%; gate remains functional.
- $\sigma_{H}$ **feedback (**±20%**):** natural 44--60%; commitment timing shifts ±25min.

The qualitative Fujita asymmetry is preserved across all perturbations without refitting.

#### 6.2.8. Comparison with classical approaches.

**FBA:** Cannot predict threshold—lacks regulatory control, optimizes flux without commitment semantics.

**Boolean networks:** Cannot predict threshold—treats ATP as external parameter, requires threshold as input rather than output.

**Hybrid PN:** Cannot predict threshold—separates metabolism (continuous) and regulation (discrete) without unified species, forcing artificial ATP duplication.

**Classical Bio-PN with test arcs:** Cannot express commitment—test arcs are read-only, cannot model signal consumption creating irreversibility.

**Signal hierarchical PN:** Reproduces Fujita 52% vs. ≈5% asymmetry from $\sigma_{H}$ separatrix architecture, zero parameters adjusted to match this outcome.

### 6.3. Developmental scope and pre-print record

During the development of signal hierarchy theory, the formalism was applied to two further biological systems. A predecessor analysis of lambda phage lysis-lysogeny, using an information-theoretic approach that preceded the full SHPN arc-type semantics, is available as a pre-print [23] (arXiv:2512.22415, submitted December 2025). An earlier 13-tuple formulation applied to *V. fischeri* quorum sensing is available as a pre-print [24] (arXiv:2601.00036, posted January 2026). Neither is presented as SHPN validation here: both pre-dates the signal-consumption semantics formalized in this paper, and both are being revised under the mature definition before formal journal submission. A GATA1/PU.1 haematopoietic lineage commitment model applying the same formalism is in active preparation (E.S., unpublished). These developmental works establish breadth of applicability and are noted here to avoid duplication should readers encounter the pre-prints independently.

## 7. Methods

Stochastic sweep simulations use a τ-leaping engine; deterministic simulations use an ODE solver with Hill-function kinetics. Both engines are implemented in the SHPN simulator (source code: https://github.com/simao-eugenio/shypn, https://doi.org/10.5281/zenodo.21478129). All signal arc parameters (Γ=(K,n,ε), θ, $W_{s}$) are drawn from published sources (BRENDA, Voet & Voet [21]); no parameter was adjusted to match the Fujita efficiency fractions. Complete model specifications, rate constants, initial conditions, and sensitivity analysis are documented in [25].

## 8. Discussion

Signal hierarchical Petri nets establish topology-driven threshold computation through four integrated innovations: signal hierarchy theory proving structural theorems, signal hierarchical control formalizing preemption mechanisms, 13-tuple formalism extending Bio-PN expressiveness, and unified metabolic-regulatory modeling enabling modeler-designated signal places. This progression—theory to control to formalism to application—addresses fundamental gap in computational biology: existing frameworks treat metabolites either as passive substrates or as Boolean parameters, never as modeler-designated species with differentiated arc semantics.

A clarification on feedback and acyclicity is warranted. The acyclicity requirement applies exclusively to $G_{s}=(\Psi ,F_{s})$, the signal control subgraph. The execution graph $G_{E}=(P,T,F)$ is entirely unrestricted: it may contain cycles, supporting metabolic feedback inhibition, oscillatory dynamics, and recycling loops — all biological realities. Feedback regulation is modeled in $G_{E}$ through normal arcs and in Φ through rate functions that may reference any place in any direction. What the acyclicity requirement excludes is a directed cycle *through hierarchical commitment arcs* $F_{s}$: such a cycle would mean the cell could uncommit itself through the same channel that committed it, which has never been observed. In this sense SHPN extends Petri’s partial order semantics [26]: where occurrence nets establish acyclicity in the *causal execution record* (the unfolding of a net’s behavior over time [27]), SHPN establishes acyclicity in the *control topology itself* — not as a property of any particular execution history, but as a structural constraint on the commitment architecture. The genome ($G_{E}$) contains all possible epigenetic states at high entropy; the commitment topology ($G_{s}$) is the irreversible selector that collapses those possibilities once a threshold is crossed.

### 8.1. Theoretical contributions

**Signal hierarchy theory** advances Petri net foundations by proving two structural properties distinguishing hierarchical commitment from general regulation. The acyclicity theorem formalizes hierarchical stratification for connected commitment channels: metabolism precedes sensing precedes integration precedes execution through signal flow arcs, forbidding circular preemption dependencies where committed decisions create their own enabling conditions. Importantly, this constraint applies only to topology-explicit connected structure ($F_{s}$ arcs defining preemption), not to equation-implicit remote sensing (Φ enabling feedback through kinetic dependencies). The preemption theorem establishes vertical dependencies: lower-layer threshold failures propagate upward through connected channels, disabling higher layers regardless of local token abundances. These theorems codify the distinction between hierarchical commitment (must be stratified, acyclic connected paths) and feedback regulation (can be cyclic, remote sensing without preemption coupling), enabling formal verification of hierarchical correctness while preserving regulatory feedback capabilities.

**Consumptive information semantics** differentiate signal flow arcs from test arcs, resolving expressiveness limitations of classical Bio-PN. Test arcs model catalysis (enzymes reading substrate concentrations without consumption), signal flow arcs model commitment (decision systems depleting regulatory quotas during irreversible choices). This semantic distinction—read-only vs. consumptive—determines predictive capability: test-arc models require experimental threshold measurements, signal-flow models compute thresholds from topology. The innovation: information can be consumed like mass but transferred like signals, creating hybrid entity (regulatory tokens) enabling threshold computability.

**Basin boundary computability** establishes thresholds as structural properties. Classical dynamical systems require simulation to find attractor basins—computationally expensive state space exploration. Signal hierarchical networks provide closed-form formula: $M_{\text{commit}}=\theta +W_{s}$. No simulation, no fitting, no iteration—extract parameters from literature, apply formula, obtain quantitative prediction. This reduces threshold determination from Ackermann-complete reachability problem [19] to *O*(1) arithmetic, enabling genome-scale analyses infeasible with simulation-based methods.

Relationship to related formalisms: Beyond classical Bio-PN, SHPN relates to a broader tradition of process-oriented and partial-order-based approaches. Condition/Event nets (C/E nets [28]) distinguish conditions (Boolean state) from events (occurrences), encoding the non-consuming vs. consuming distinction at the token level—the closest precursor to the test-arc / signal-arc distinction in SHPN. Occurrence nets and net unfoldings [26,27] establish acyclicity in the causal execution record, capturing the irreversibility of events in the temporal unfolding of concurrent processes. Process-algebraic approaches (Regev and Shapiro [29], Priami [30]) model individual molecular interactions concurrently through process composition, excelling at combinatorial signaling and competitive binding. These traditions address complementary aspects: process algebras model concurrent interaction identity; occurrence nets model causal irreversibility in execution history; SHPN models population-level commitment thresholds through a structural constraint on the *control topology itself*. In Petri’s partial order tradition, acyclicity is a property of the execution record (the unfolding is always a DAG even if the underlying net has cycles). SHPN adds a second, independent acyclicity requirement: $G_{s}$ must be a DAG as a condition on the net structure—because biological commitment channels are irreversible by design, not merely by the history of one particular run.

### 8.2. Methodological advances

**Unified metabolic-regulatory modeling** addresses the architectural separation of metabolic and regulatory concerns in classical systems biology approaches. FBA optimizes metabolism ignoring regulation. Boolean networks model regulation treating metabolites as parameters. Hybrid models separate concerns with artificial interfaces. Signal hierarchical PN unifies through modeler designation: any metabolite becomes signal place $p_{s}\in \Psi$ when biological function supports it, participating in biochemical networks (normal arcs, horizontal mass transfer) and regulatory hierarchies (signal flow arcs, vertical information propagation) simultaneously. No duplication, no parameterization, no separation—integrated species with differentiated arc semantics. For *B. subtilis*, we designate $\sigma_{H}$ as the commitment-gate signal and Spo0A~P as the phosphorelay integration signal; for calcium signaling, Ca^2+^ is designated; for chemotaxis, cAMP. This architectural unification enables analyses impossible in fragmented frameworks: How does metabolic perturbation propagate through sensing layers to alter commitment thresholds? Unified model answers directly; fragmented models require manual integration.

**Topology-driven threshold computation** distinguishes this work from parameter-fitting approaches. Classical workflow: build model, measure thresholds experimentally, fit parameters, validate. Signal hierarchical workflow: designate signals from biological evidence, extract arc parameters from published biochemical constants, compute $M_{\text{commit}}=\theta +W_{s}$, compare to independent experimental data. The structural inversion: the threshold is an output of the topology, not an input to a fit. For *B. subtilis* sporulation, the $\sigma_{H}$ separatrix at ≈1.60μM accounts for the Fujita and Losick outcome asymmetry; the Γ arc parameters were drawn from independent biochemical data, not tuned to that ratio. This distinction matters for interpretation: a correctly parameterized mechanistic model is *expected* to reproduce experimental observations, because correct parameters reflect real molecular properties. The match is therefore not coincidental — it is evidence that the parameterization is correct. What makes it a genuine validation rather than a tautology is that the Fujita ratio was never consulted during model construction: the 52%/5% asymmetry could not have been obtained by other means without also capturing the correct $\sigma_{H}$ separatrix and phosphorelay dynamics.

**Hierarchical decomposition** ameliorates computational complexity. Reachability in Petri nets is Ackermann-complete [19], limiting classical methods to small models (<50 places). Signal hierarchy enables divide-and-conquer: analyze Layer 0 independently (metabolic production of designated signals), compute commitment thresholds, use thresholds as Layer 1 constraints, analyze sensing dynamics, repeat upward. The complexity reduces from exponential product $\prod_{i}|M_{{\text{Layer}}_{i}}|$ over layers to linear sum $\sum_{i}|M_{{\text{Layer}}_{i}}|\cdot b_{i}$ times branching factors. *B. subtilis* model (40 places, 4 layers) analyzed in 2.3 seconds; equivalent flat model requires estimated 10 + hours. Hierarchical structure converts intractable problem into practical computation.

### 8.3. Biological insights

**Signal designation as gatekeeper:** Modeler-designated signals create biological firewalls preventing infeasible commitments. For *B. subtilis*, $\sigma_{H}$ is designated as the commitment-gate signal (Layer 2). Commitment without crossing the $\sigma_{H}$ separatrix ($\theta_{\sigma H}\approx 1.60\;\mu$M) is structurally impossible: the Preemption theorem disables all downstream execution layers ($\sigma_{F}$, $\sigma_{E}$, $\sigma_{G}$, $\sigma_{K}$) regardless of Spo0A~P abundance. Biology implements a fail-safe: the commitment gate vetoes execution when the gating signal is insufficient, preventing premature or incomplete developmental programs. This principle generalizes: Ca^2+^ depletion disables neurotransmitter release, GTP depletion halts protein synthesis, cAMP depletion blocks chemotaxis—all through the same hierarchical preemption mechanism applied to different designated signals.

**Irreversibility through consumption:** Commitment creates one-way transitions via signal depletion. Sporulation initiation consumes regulatory tokens (activated Spo0A~P), establishing basin boundary: pre-commitment cells (below threshold) occupy reversible attractor basin; post-commitment cells (above threshold, fired) occupy irreversible basin. No firing sequence reverses—signal consumption prevents return. This explains experimental observations: committed cells complete sporulation even when nutrients return (irreversibility); uncommitted cells return to vegetative growth when nutrients restored (reversibility). Mathematical model captures biological finality through consumptive semantics.

**Hierarchical signal integration:** Multi-layer architecture enables cascading irreversibility (Section 5.2): each layer establishes its own basin boundary via signal consumption, and depletion at any layer propagates upward through PreemptionCheck to disable all higher layers. This stratification enables robust decisions: single sensor failure does not prevent commitment (redundancy), but designated signal collapse does (preemption). Hierarchy balances reliability (multiple pathways) with safety (designated resource gating). The architecture generalizes: calcium signaling designates Ca^2+^, chemotaxis designates cAMP, transcription quality control designates GTP—same hierarchical organization, different signal identities.

### 8.4. Limitations and future directions

**Spatial heterogeneity:** Current formalism assumes well-mixed compartments. Bacterial sporulation exhibits spatial asymmetry: mother and forespore compartments differ in regulatory states. Extending signal hierarchical PN with compartmentalization (colored tokens, compartment-specific transitions) would enable spatial commitment modeling. Challenge: maintaining threshold computability when diffusion creates continuous spatial gradients.

**Stochastic dynamics and noise analysis:** The hybrid formalism supports both deterministic and stochastic simulation engines, including tau-leaping and Stochastic Simulation Algorithm (SSA) implementations enabling efficient stochastic parallelism. For the *B. subtilis* case study, deterministic kinetics suffice for threshold prediction because commitment thresholds represent mean-field basin boundaries—population-level decision points independent of single-cell trajectory noise. However, stochastic engines become essential when analyzing commitment reliability under low-copy-number fluctuations (e.g., transcription factor noise affecting decision timing) or computing commitment probability distributions. The challenge: extending topology-based threshold formulas $M_{\text{commit}}=\theta +W_{s}$ to stochastic settings where basin boundaries become probability distributions rather than deterministic values. Future work includes developing analytical approximations for commitment probability as function of signal variance.

**Parameter uncertainty:** Literature parameters have experimental error (±20-50%). Current sensitivity analysis shows robustness to individual parameter variations, but systematic uncertainties require probabilistic treatment. Bayesian parameter estimation over signal hierarchical models would quantify prediction confidence intervals. Challenge: maintaining topology-driven philosophy when incorporating posterior distributions from data.

**Genome-scale models:** *B. subtilis* model has 40 places; genome-scale metabolic models exceed 1000 metabolites. Hierarchical decomposition enables scaling, but manual layer assignment becomes infeasible. Developing automated layer inference algorithms (e.g., graph partitioning optimizing vertical-horizontal separation) would enable genome-scale signal hierarchical models. Challenge: biological hierarchy not always obvious from network topology alone.

**Experimental validation:** Current validation compares predicted thresholds to published measurements. Stronger validation: experimentally perturb predicted parameters (e.g., engineer ATP synthase mutants), predict shifted thresholds, measure in vivo. Prospective predictions (before measurement) provide more rigorous tests than retrospective comparisons (after measurement).

### 8.5. Applications

**Synthetic biology:** Designing decision circuits with predictable commitment thresholds requires the threshold to be computable from the circuit topology before construction. Current practice: build circuit, measure threshold, iterate. Signal hierarchical approach: specify desired threshold, compute required signal weights backward ($W_{s}=M_{\text{commit}}-\theta$), construct circuit matching specifications.

**Drug discovery:** ATP-dependent pathways represent therapeutic targets. Cancer cells commit to proliferation at higher ATP thresholds than normal cells, creating therapeutic window. Signal hierarchical models predict: (1) which pathways exhibit ATP-dependent thresholds, (2) threshold values for normal vs. cancer cells, (3) compounds shifting thresholds (ATP synthase inhibitors). Enables prioritizing targets with maximum differential between cell types.

**Patient-specific parameterisation:** Signal hierarchical models can, in principle, be instantiated from patient-specific enzyme and metabolite measurements to compute individualised commitment thresholds. This direction requires experimental validation of the topology-first assumption across heterogeneous populations and is reserved as future work.

**Systems biology:** Understanding cellular decision-making requires integrated metabolic-regulatory models. Signal hierarchical PN enables: (1) computing how metabolic perturbations (nutrient shifts) alter commitment thresholds, (2) analyzing commitment reliability under energetic fluctuations, (3) designing experiments probing basin boundaries. Broader impact: framework applicable beyond bacteria—mammalian cell cycle, immune cell differentiation, stem cell fate decisions all exhibit energy-gated commitment with predictable thresholds.

## 9. Conclusions

We establish signal hierarchy theory as formal foundation for hierarchical biological control, implement theory as signal hierarchical Petri nets extending Bio-PN from 5-tuple to 13-tuple formalism, and demonstrate mechanistic threshold prediction through *Bacillus subtilis* sporulation case study approximately reproducing the Fujita and Losick [14] experimental observation (48−−52% natural vs. ≈5% abrupt sporulation) with no arc parameter optimized to match these fractions. Four original contributions advance computational biology:

1. **Signal hierarchy theory** proves structural theorems establishing acyclicity (hierarchical stratification forbidding circular control) and preemption (lower-layer depletion disabling higher layers), formalizing biological principles as mathematical necessities.
2. **Signal hierarchical control** implements theory through consumptive information semantics distinguishing signal flow arcs (regulatory quota depletion) from test arcs (catalytic reading), enabling irreversible commitment modeling impossible in classical Bio-PN.
3. **Signal hierarchical Petri nets** provide 13-tuple formalism with dual arc semantics enabling unified metabolic-regulatory modeling where any metabolite may function simultaneously as biochemical substrate (via normal arcs) and designated regulatory signal (via signal flow arcs): in the *B. subtilis* case, $\sigma_{H}$ gates sporulation commitment while also being produced and consumed by the metabolic network.
4. **Unified framework** enables topology-driven threshold computation: $M_{\text{commit}}=\theta +W_{s}$ computable from literature parameters without simulation or fitting, reducing Ackermann-complete reachability [19] to *O*(1) arithmetic.

Mathematical validation through proven theorems establishes correctness. Biological validation on *B. subtilis* sporulation demonstrates that the $\sigma_{H}$ separatrix threshold computed from the arc-level quantities θ and $W_{s}$ reproduces the Fujita and Losick experimental outcome asymmetry without parameter fitting. Predecessor applications to lambda phage and *V. fischeri* quorum sensing, cited as pre-prints [23,24], establish developmental breadth and will be formally published under the mature semantics.

## References

[1] Orth JD, Thiele I, Palsson BØ. What is flux balance analysis?. Nat Biotechnol. 2010;28(3):245–8. doi: 10.1038/nbt.1614 20212490 PMC3108565

[2] Kauffman SA. Metabolic stability and epigenesis in randomly constructed genetic nets. J Theor Biol. 1969;22(3):437–67. doi: 10.1016/0022-5193(69)90015-0 5803332

[3] Liu B, Hagiescu A, Palaniappan SK, Chattopadhyay B, Cui Z, Wong W-F, et al. Approximate probabilistic analysis of biopathway dynamics. Bioinformatics. 2012;28(11):1508–16. doi: 10.1093/bioinformatics/bts166 22492313

[4] Alla H, David R. Continuous And Hybrid Petri Nets. J Circuit Syst Comp. 1998;08(01):159–88. doi: 10.1142/s0218126698000079

[5] Petri CA. Kommunikation mit automaten. University of Bonn. 1962.

[6] Murata T. Petri nets: Properties, analysis and applications. Proc IEEE. 1989;77(4):541–80. doi: 10.1109/5.24143

[7] Reddy NR, Mavrovouniotis ML, Liebman MN. Petri net representations in metabolic pathways. In: Proceedings of the First International Conference on Intelligent Systems for Molecular Biology (ISMB), 1993. 328–36. 7584354

[8] Heiner M, Gilbert D, Donaldson R. Petri Nets for Systems and Synthetic Biology. Lecture Notes in Computer Science. Springer Berlin Heidelberg. p. 215–64. doi: 10.1007/978-3-540-68894-5_7

[9] Chaouiya C. Petri net modelling of biological networks. Brief Bioinform. 2007;8(4):210–9. doi: 10.1093/bib/bbm029 17626066

[10] Reisig W. Petri Nets: An Introduction. Berlin: Springer-Verlag. 1985.

[11] Koch I, Reisig W, Schreiber F. Modeling in systems biology: The Petri net approach. London: Springer. 2011.

[12] Matsuno H, Tanaka Y, Aoshima H, Doi A, Matsui M, Miyano S. Biopathways representation and simulation on hybrid functional Petri net. In Silico Biol. 2003;3(3):389–404. 12954096

[13] Goss PJ, Peccoud J. Quantitative modeling of stochastic systems in molecular biology by using stochastic Petri nets. Proc Natl Acad Sci U S A. 1998;95(12):6750–5. doi: 10.1073/pnas.95.12.6750 9618484 PMC22622

[14] Fujita M, Losick R. Evidence that entry into sporulation in Bacillus subtilis is governed by a gradual increase in the level and activity of the master regulator Spo0A. Genes Dev. 2005;19(18):2236–44. doi: 10.1101/gad.1335705 16166384 PMC1221893

[15] Heiner M, Koch I, Will J. Model validation of biological pathways using Petri nets--demonstrated for apoptosis. Biosystems. 2004;75(1–3):15–28. doi: 10.1016/j.biosystems.2004.03.003 15245801

[16] Koch I, Junker BH, Heiner M. Application of Petri net theory for modelling and validation of the sucrose breakdown pathway in the potato tuber. Bioinformatics. 2005;21(7):1219–26. doi: 10.1093/bioinformatics/bti145 15546934

[17] Gilbert D, Heiner M. A model checking approach to the analysis of biological systems. Computational Methods in Systems Biology. 2006;4210:122–40.

[18] Baldan P, Corradini A, Montanari U. Contextual Petri nets, asymmetric event structures, and processes. Theoretical Computer Science. 2001;273(1–2):223–48.

[19] Czerwinski W, Orlikowski L. Reachability in Vector Addition Systems is Ackermann-complete. In: 2021 IEEE 62nd Annual Symposium on Foundations of Computer Science (FOCS), 2022. 1229–40. 10.1109/focs52979.2021.00120

[20] Schomburg I, Chang A, Ebeling C, Gremse M, Heldt C, Huhn G, et al. BRENDA, the enzyme database: updates and major new developments. Nucleic Acids Res. 2004;32(Database issue):D431-3. doi: 10.1093/nar/gkh081 14681450 PMC308815

[21] Voet D, Voet J, Pratt C. Biochemistry. 5th ed. Hoboken, NJ: Wiley. 2016.

[22] Barbe V, Cruveiller S, Kunst F, Lenoble P, Meurice G, Sekowska A, et al. From a consortium sequence to a unified sequence: the Bacillus subtilis 168 reference genome a decade later. Microbiology (Reading). 2009;155(Pt 6):1758–75. doi: 10.1099/mic.0.027839-0 19383706 PMC2885750

[23] Simão E. Hierarchical preemption: A novel information-theoretic control mechanism in lambda phage decision-making. arXiv preprint. 2024. doi: 10.48550/arXiv.2512.22415

[24] Simão E. Unifying weak independence and signal hierarchy theory: Extended biological petri net formalism with application to Vibrio fischeri quorum sensing. arXiv preprint. 2025. doi: arXiv:2601.00036

[25] Simão E. Thermodynamic constraints drive hierarchical preemption in cellular decision-making: A hybrid petri net framework with application to Bacillus subtilis sporulation. arXiv preprint. 2026. doi: arXiv:2601.04335

[26] Nielsen M, Plotkin G, Winskel G. Petri nets, event structures and domains, part I. Theoretical Computer Science. 1981;13(1):85–108. doi: 10.1016/0304-3975(81)90112-2

[27] Winskel G. Event structures. Lecture Notes in Computer Science. Springer Berlin Heidelberg. 1987. p. 325–92. doi: 10.1007/3-540-17906-2_31

[28] Rozenberg G, Engelfriet J. Elementary net systems. Lecture Notes in Computer Science. Springer Berlin Heidelberg. 1998. p. 12–121. doi: 10.1007/3-540-65306-6_14

[29] Regev A, Shapiro E. Cells as computation. Nature. 2002;419(6905):343. doi: 10.1038/419343a 12353013

[30] Priami C. Stochastic pi-calculus. The Computer Journal. 1995;38(6):578–89.

