# Supplementary material for: Signal hierarchical petri nets: Formal semantics of hierarchical regulatory control of biological systems
Source: PLoS One. 2026 Aug 21;21(8):e0355540. doi: 10.1371/journal.pone.0355540 (PMC13496463; doi:10.1371/journal.pone.0355540)
Supplement: Supplementary file 1 [file pone.0355540.s001.pdf]

Referee Report

Referee Report on "Signal Hierarchical Petri Nets: Formal Semantics of Hierarchical Regulatory Control of Biological Systems"

Summary

This manuscript introduces *Signal Hierarchical Petri Nets* (SHPN), a 13-tuple extension of classical Biological Petri Nets grounded in what the author terms *Signal Hierarchy Theory.* The central idea is to distinguish between horizontal mass-transfer arcs and vertical consumptive signal-flow arcs, thereby enabling unified metabolic–regulatory modeling within a single formalism. The framework establishes structural theorems (acyclicity, hierarchical preemption, irreversible basin partitioning) and proposes that biological commitment thresholds can be computed structurally via the closed-form expression

$$M_{\text{commit}} = \theta(t) + W_s((p_s, t)).$$

The approach is illustrated through a case study of *Bacillus subtilis* sporulation, where the predicted ATP commitment threshold closely approximates experimental values. The rigor is very good. However, the theorems are what we should expect from the definitions. They have the main purpose of justifying the definitions, being a kind of mathematical retionale. This is well-done.

Major Strengths

1. **Conceptual originality.** The introduction of consumptive signal semantics represents a clear conceptual advance beyond classical Bio-PN and test-arc extensions. The distinction between mass transfer and signal consumption is well articulated and mathematically precise.

2. **Formal rigor.** The manuscript provides explicit definitions, enablement predicates, firing rules, and structural theorems. The acyclicity and hierarchical preemption results are logically coherent and appropriately proven.

3. **Unified modeling of metabolism and regulation.** Allowing the same place to participate in both metabolic and regulatory roles through arc-type assignment is biologically motivated and technically elegant. This avoids artificial duplication of species often seen in hybrid approaches.

4. **Closed-form threshold computation.** The structural commitment formula is simple, interpretable, and potentially impactful. Turning commitment thresholds into computable topological properties is a noteworthy contribution.

5. **Illustrative biological validation.** The *B. subtilis* sporulation example is carefully developed and includes sensitivity analysis, strengthening the plausibility of the framework.

Major Concerns

1. **Scope of "first-principles" claim.** While thresholds are computed structurally, the parameters $\theta(t)$ and W_s are derived from experimentally measured biochemical constants. The manuscript would benefit from clarifying that the prediction is topology-driven but parameter-dependent, rather than entirely parameter-free.

2. **Biological generality.** The main detailed validation focuses on a single biological system. Although additional examples are mentioned, they are not fully developed. For a generalist journal such as *PLOS ONE*, broader empirical validation or computational benchmarking across multiple systems would strengthen the manuscript.

3. **Acyclicity assumption.** The requirement that signal flow graphs be acyclic is theoretically justified, but many biological regulatory networks exhibit feedback loops. The manuscript addresses this through remote sensing via rate functions Φ, yet the conceptual separation between "connected" and "remote" regulation may require further clarification to avoid the impression of biological oversimplification.

4. **Comparison with existing extended PN frameworks.** A more systematic comparison with alternative Petri net extensions (e.g., inhibitor arcs, colored PN, hybrid PN variants) would help situate the contribution within the broader modeling literature. There are approaches defined on top of universal semantic principles, using Petri-Nets, that models the BioMolecular realm as a Turing Machine models the symbolic computational realm, via Turing-Church Thesis (Hypothesis). They do not separate signals and mass, so that a bit more of fundamental explaining would be welcome, since they catch the Intensional Semantics of the BioMolecular processes, without needing external paprameters.

Minor Comments

- The terminology is dense and layered. A high-level schematic diagram early in the manuscript summarizing the dual-graph architecture would improve accessibility.

- Some sections could be streamlined to reduce repetition between theoretical exposition and biological discussion.

Recommendation

This manuscript presents a mathematically rigorous and conceptually innovative extension of Biological Petri Nets with potential relevance for systems biology modeling. The theoretical contribution is substantial and internally consistent. However, clarification of the "first-principles" claim and expanded biological validation would significantly strengthen the work for publication in *PLOS ONE*.

Subject to satisfactory revision addressing the concerns above, I consider the manuscript suitable for publication. The edition and presentation is ok. However, the author should observe our observations and suggestions on the content and foundational principles.
