# Supplementary material for: Signal hierarchical petri nets: Formal semantics of hierarchical regulatory control of biological systems
Source: PLoS One. 2026 Aug 21;21(8):e0355540. doi: 10.1371/journal.pone.0355540 (PMC13496463; doi:10.1371/journal.pone.0355540)
Supplement: Supplementary file 2 [file pone.0355540.s003.pdf]

Response to Reviewers
Manuscript: PONE-D-26-05900
Title: Signal Hierarchical Petri Nets: Formal Semantics of Hierarchical Regulatory Control of Biological Systems
Date: June 22, 2026

We thank the editor and all three reviewers for their careful and constructive evaluation. The reviewers' comments have substantially improved the manuscript, and we are grateful for the opportunity to revise. Below we address each point raised, in the order: Editor, Reviewer 1 (Prof. Haeusler), Reviewer 2 (Dr. Kolčák), Reviewer 3.

Inline revision references of the form [**Revision: Section X, para. Y**] indicate where each change can be located in the revised manuscript. A track-changes PDF is submitted alongside this letter.

Editor (Dr. Jianhong Zhou)

> *The reviewers have raised a number of concerns that need attention, especially the contradictory claims. In addition, please disclose if there is any artificial intelligence (AI) tools and technologies usage following our policy.*

On contradictory claims. The principal contradiction identified by the reviewers — that the manuscript at times claims "first-principles" (parameter-free) threshold computation while simultaneously acknowledging that the parameters θ and W_s are derived from experiment — has been corrected throughout the revised manuscript. The phrase "first-principles prediction" has been replaced in all ten occurrences by "topology-driven threshold computation" or equivalent phrasing that makes the parameter-dependence explicit. A clarifying sentence has been added wherever the threshold formula appears: *The parameters θ and W_s are derived from experimentally measured biochemical constants; the structural claim is that the threshold is localized to at most two quantities per signal arc, rather than emerging from a global nonlinear fit of the whole system.*

A second contradictory claim — that SHPN provides "unified semantics, as opposed to a hybrid Petri net approach" while the formal definition is an extension of a hybrid Petri net — has also been corrected. The revised Introduction and Section 4 now state that SHPN is a hybrid Petri net in the established sense of the term (coexistent continuous and stochastic dynamics) but extends the classical hybrid PN formulation by adding a unified signal-type layer. [**Revision: Introduction, final paragraph; Sec. 4.1, Definition 2 preamble.**]

On AI tools. The revised manuscript includes an explicit disclosure paragraph in the Acknowledgments section, consistent with PLOS ONE policy. The paragraph reads: *"The author used AI-assisted tools (GitHub Copilot) as a collaborative aid for drafting, editing, and formulating arguments. All scientific insights — the formalism, the theorems and their proofs, the biological interpretations, and the experimental comparisons — originate entirely from the author. The AI tools served to sharpen prose; they did not generate scientific content. This manuscript was written with genuine intellectual engagement and the author accepts full scientific responsibility for all claims."*

On Journal Requirements (items 1–4). Style requirements are followed. The data availability statement will be updated as requested; see our response to Phase 4 items. Both Table 1 and Table 2 are now referred to explicitly in the text. [**Revision: Sec. 3.2, after Table 1; Sec. 4.4, after Table 2.**] Reviewer-recommended citations have been evaluated; those relevant to the scope of the paper have been added.

Reviewer #1 (Prof. Edward Hermann Haeusler)

We thank Prof. Haeusler for his generous assessment and his expert framing of the contribution. His comments guided the most substantive improvements in the revision.

Major Concern 1 — Scope of "first-principles" claim

> *While thresholds are computed structurally, the parameters $\theta(t)$ and W_s are derived from experimentally measured biochemical constants. The manuscript would benefit from clarifying that the prediction is* topology-driven *but* parameter-dependent, *rather than entirely parameter-free.*

We agree entirely, and the revision adopts the reviewer's framing wholesale. "First-principles prediction" has been replaced throughout by "topology-driven threshold computation." The claim is now stated precisely: the threshold is *structurally localized* to the two arc-level parameters θ and W_s of the signal arc $(p_s, t) \in F_s$. Once the network topology is fixed, the threshold $M_{\text{commit}} = \theta + W_s$ is determined by at most two measurable quantities per commitment arc, rather than requiring a global nonlinear parameter fit of the full ODE system. The parameters themselves are experimentally derived; the structural contribution is the localization. [**Revision: Abstract; Introduction para. 3; Corollary 1 proof; Conclusion.**]

Major Concern 2 — Biological generality

> *The main detailed validation focuses on a single biological system. Although additional examples are mentioned, they are not fully developed. For a generalist journal such as PLOS ONE, broader empirical validation or computational benchmarking across multiple systems would strengthen the manuscript.*

We thank the reviewer for this concern. After careful consideration, we have chosen a different response than adding new systems in equal depth, for reasons of scientific accuracy.

During the development of signal hierarchy theory, earlier versions of the formalism were applied to two further biological systems:

(i) Lambda phage lysis-lysogeny switch. An information-theoretic analysis of the lambda phage decision, using a predecessor formalism that preceded the full SHPN arc-type semantics, is available as a pre-print: Simão, E. (2024) "Hierarchical Preemption: A Novel Information-Theoretic Control Mechanism in Lambda Phage Decision-Making," arXiv:2512.22415.

(ii) *Vibrio fischeri* quorum sensing. An application of an earlier 13-tuple extended Bio-PN formulation is available as a pre-print: Simão, E. (2025) "Unifying Weak Independence

and Signal Hierarchy Theory:
Extended Bio-PN Formalism with Application to *V. fischeri* Quorum Sensing," arXiv:2601.00036.

Both pre-prints pre-date the signal-consumption semantics formalised in the present paper and were deliberately not submitted for peer review until the underlying formalism reached its current form. They are cited in the revision (Sec. 5.3) as part of the developmental record. We do not present them as SHPN validations because the arc-type semantics and formalism they use differ from the definition in this paper; both are being revised under the mature semantics before formal journal submission.

The reason for restricting quantitative validation to *B. subtilis* is that only this system has an independent experimental benchmark — the Fujita and Losick (2005) 52% vs. ≈ 5% asymmetry — against which the topology-driven threshold prediction can be tested without fitting. Citing pre-print results under an earlier formalism as co-equal validation would overstated the present contribution. We believe citing the pre-prints as developmental context, while restricting validated claims to B. subtilis, is the scientifically correct position. **[Revision: New Sec. 5.3 "Developmental Scope and Pre-print Record," p. 40 of the revision.]**

Major Concern 3 — Acyclicity and feedback loops

> *The requirement that signal flow graphs be acyclic is theoretically justified, but many biological regulatory networks exhibit feedback loops. The manuscript addresses this through remote sensing via rate functions Φ, yet the conceptual separation between "connected" and "remote" regulation may require further clarification.*

We thank the reviewer for raising this point; it reflects a genuine conceptual distinction that the submitted version did not express clearly enough.

The revision clarifies this in two places. First, a dedicated paragraph in Section 3.2 (after Table 1) explains that Φ and G_S operate over *different* graph layers and answer different questions: rate functions Φ express continuous, potentially cyclic kinetics over G_E; signal arcs F_s express discrete, irreversible commitment steps over G_S. The acyclicity requirement applies *only* to G_S; G_E is entirely unrestricted and may contain the feedback loops and oscillatory circuits common in metabolic and signalling networks.

Second, the Discussion (Sec. 6) opens with a paragraph that makes this explicit: the two feedback loops that concern the reviewer — metabolic recycling and regulatory oscillation — both reside in G_E. What the acyclicity of G_S excludes is a closed directed path through *commitment arcs*, which would mean the controller can reset through its own commitment channel. No such cycle has been observed in biological commitment decisions to the authors' knowledge.

We also wish to offer a sharper biological grounding for this point. Acyclicity of G_S is not an assumption we impose — it is a *formal consequence* of the empirical observation that committed cells do not de-differentiate through the same biochemical channel that committed them. A directed cycle in G_S would require the existence of a firing sequence that regenerates, through the commitment arc itself, a signal token that was consumed in the act of deciding. This is the formal statement that a butterfly can return to its larval state through the same metamorphic channel — an event with no biological counterpart. The formalism is therefore *constructed to honour* that observation, not to impose an arbitrary

restriction. G_E cycles remain completely unrestricted because metabolic feedback and regulatory oscillation do not constitute commitment reversal — they are continuous kinetics within an already-differentiated cell, not de-differentiation events through a committed channel. [**Revision: Sec. 3.2, after Table 1 (new paragraph); Sec. 6, opening paragraph.**]

Major Concern 4 — Comparison with existing PN frameworks

> *A more systematic comparison with alternative Petri net extensions would help situate the contribution. There are approaches defined on top of universal semantic principles, using Petri-Nets, that models the BioMolecular realm as a Turing Machine models the symbolic computational realm, via the Turing-Church Thesis. They do not separate signals and mass, so that a bit more of fundamental explaining would be welcome, since they catch the Intensional Semantics of the BioMolecular processes, without needing external parameters.*

This is a well-taken point, and we are grateful to Prof. Haeusler for indicating the intensional-semantics tradition. The revision adds a dedicated paragraph in Section 4.4 (*Comparison with Classical Bio-PN*) that situates SHPN relative to four traditions:

- *C/E nets and occurrence nets* (Rozenberg 1998; Nielsen, Plotkin, Winskel 1981): these formalisms capture causal dependence and concurrency without introducing mass. SHPN inherits their partial-order semantics for the G_S layer but adds the mass-transfer layer G_E to handle stoichiometric species concentrations.
- *Process algebras* (Regev & Shapiro 2002; Priami 2005): π-calculus and stochastic π-calculus model molecular interaction through channel communication, capturing the intensional semantics the reviewer refers to. These approaches are complementary: they excel at mobile processes and communication topology; SHPN addresses commitment thresholds as structural properties of a fixed network.
- *Classical hybrid PN* (Alla & David 1998; Matsuno 2003): SHPN extends this tradition by adding unified signal-type semantics. The coexistence of continuous and stochastic dynamics is inherited; the G_S/acyclicity layer is new.

Regarding the intensional-semantics tradition specifically: we agree that formalisms based on the Turing-Church thesis for biomolecular computation provide a principled account of cellular processes without requiring arc-type distinctions. Our approach is a different trade-off: SHPN sacrifices some generality (we require explicit arc-type assignment) in exchange for a structural theorem that makes commitment thresholds computable from the topology. Neither approach is more fundamental; they answer different questions. [**Revision: Sec. 4.4, new comparison paragraph with citations.**]

Minor Comment — Dual-graph diagram

> *A high-level schematic diagram early in the manuscript summarizing the dual-graph architecture would improve accessibility.*

A TikZ figure (Fig. 1, *Dual-graph architecture of SHPN*) has been added at the close of the Introduction. It shows G_E (left panel, with normal arcs, signal places, and a feedback cycle) and G_S (right panel, as a DAG with layer depths $\lambda = 0$ and $\lambda = 1$) side by side, connected

by a bridge indicating that the signal place is shared between both graphs. [**Revision: End of Introduction, new Fig. 1.**]

Minor Comment — Streamlining

> *Some sections could be streamlined to reduce repetition between theoretical exposition and biological discussion.*

We have reduced repetition in Sections 3 and 5 by removing or condensing passages that re-stated definitions already given formally. The revision is somewhat longer than the submitted version due to the second biological example requested by this reviewer; we have tried to offset that growth by tightening existing prose elsewhere.

Reviewer #2 (Dr. Juri Kolčák)

We thank Dr. Kolčák for his detailed and precise technical critique. His formal objections were largely correct, and addressing them has made the manuscript considerably more rigorous. We respond to each point below.

We note that Dr. Kolčák recommends rejection on grounds of formal deficiencies. We believe the specific deficiencies he identified have been fully repaired in the revision. We address his broader concerns — whether Φ makes the rest of the formalism redundant, and whether SHPN introduces anything new — in dedicated subsections below.

Point 1 — T undefined in Definition 1

> *[The manuscript] immediately relies on undefined concepts (T has not been defined in Def. 1).*

Correct. The revised Definition 1 now opens with: *"Let T be the set of transitions of the underlying Petri net."* T is defined before any use. [**Revision: Sec. 2.1, Definition 1, first line.**]

Point 2 — Notation E_s vs. F_s

> *[The manuscript] disrespects its own established notation (E_s magically turns into F_s).*

The inconsistency arose from a late renaming that was not propagated everywhere. All occurrences of E_s in the submitted manuscript have been replaced with F_s, including within Theorem 1 (Acyclicity Requirement) and all body text. The notation is now consistent throughout. [**Revision: All occurrences; verified by global search.**]

Point 3 — W_t missing from the 13-tuple

> *The "formal" definition does not include all the components utilised in prior text (e.g. W_t).*

The test-arc weight W_t has been addressed by stating the convention explicitly within the F_t bullet of the 13-tuple: *"Test arc enablement requires $M(p) \geq 1$ for all $(p, t) \in F_t$ — the sensing threshold is uniformly 1 and is not a free parameter of the formalism."* The decision

not to add a new symbol W_t to the tuple is deliberate: since $W_t \equiv 1$ for all test arcs, adding it as a 14th component would introduce a symbol that carries no modelling freedom and could mislead readers into expecting a parameter to tune. The convention is now stated explicitly instead of being left implicit. [**Revision: Sec. 4.1, 13-tuple, F_t bullet.**]

Point 4 — Theorems 2 and 3: missing constraints

> *The "Hierarchical Preemption" requires extensive constraints on the shape of the undefined set of transitions T, which have never been made, nor alluded to. Essentially the same claim is made in Theorem 3, where the "irreversibility" part of the proof, which relies on those missing constraints, is simply hand-waved away.*

Both theorems now carry their necessary hypotheses explicitly in their statements (not only in the proof):

Theorem 2 now opens: *"Assuming the PreemptionCheck predicate is enforced: transition t_j is enabled only if every transition t_i that produces its signal inputs via F_s paths is itself enabled."*

Theorem 3 now states: *"Assuming no other transition regenerates p_s via F_s (i.e., there is no $t' \neq t$ with $(t', p_s) \in F_s$)"* as a hypothesis, and the proof uses "By hypothesis" instead of an aside.

We agree with the reviewer that unstated hypotheses are a formal deficiency. [**Revision: Sec. 2.2, Theorems 2 and 3.**]

Point 5 — Remote sensing (Φ) subsumes everything

> *In Section 3.2, "remote sensing" is introduced in which transitions are assigned a function that determines whether they are enabled without any regards to the network topology or constraints on the shape of the function itself. This formalism is far more rich than a classical Petri net, and makes literally all other formalisms discussed in the paper obsolete, as it subsumes them. It is also, not novel.*

This is the most substantive technical point raised by Reviewer 2, and we thank Dr. Kolčák for pressing it precisely. The submitted version did not address it adequately. We offer a response that engages with the reviewer's own research domain.

Dr. Kolčák's work on most-permissive semantics (Paulevé, Kolčák et al., *Nature Communications* 2020; Haar & Kolčák, CMSB 2025) asks: *what states can a network reach under the least restrictive interpretation?* This is the question of *maximal reachability* — the full accessible state space of G_E under the most permissive firing semantics.

G_S commitment answers the *dual question*: *what has been permanently ruled out?*

Each firing of a commitment transition t whose input arc $(p_s, t) \in F_s$ consumes tokens from signal place p_s *monotonically reduces the reachable state space of G_E*. The committed cell still carries the same genome — the uncommitted states are still topologically present in G_E — but the signal tokens that made those states accessible have been irreversibly consumed. Acyclicity of G_S guarantees this reduction is *monotone*: no subsequent G_S firing can restore the eliminated reachable states through a committed channel. The epigenetic possibility space can only narrow, never widen, through the commitment layer.

This gives a precise formal separation from Φ: rate functions provide *kinetic expressiveness* over G_E, answering the most-permissive reachability question. G_S provides *structural certificates of irreversibility*, answering the dual question. A model with unconstrained Φ and no G_S layer can simulate any trajectory — but it cannot *prove* that any trajectory is permanently excluded. G_S is the mechanism that makes irreversibility a theorem rather than a simulation outcome.

Concretely: two models with identical Φ but different arc-type assignments have different G_S graphs and therefore different monotone reachability reduction sequences. The structural theorems distinguish them; Φ alone does not. Φ is a kinetic substrate inherited from Bio-PN; SHPN's contribution is the G_S layer and the structural theorems its topology enables.

A second, limiting argument seals the point. Consider a Petri net in which all regulation is encoded in Φ and *no arcs exist at all*. Such a net has no token flow: every transition fires but consumes and produces nothing, so the marking M never departs from M_0. Φ reads the same static values forever. The model is a system of rate equations evaluated at fixed initial conditions — trivially solvable, biologically inert, structurally vacuous. The moment a signal flow arc is added, tokens are consumed on firing, the marking changes irreversibly, and the basin boundary $M_{\text{commit}} = \theta + W_s$ becomes computable from the arc topology alone. Φ expresses what a transition *reads*; only F_s arcs determine what it *destroys*. It is destruction — irreversible token consumption — that creates the one-way barrier, and that is precisely what Φ cannot do regardless of how rich its function class is.

We therefore regard the two frameworks as formally dual and genuinely complementary: most-permissive semantics establishes the ceiling of what can happen; G_S commitment establishes the floor of what has been permanently ruled out. Neither subsumes the other; they answer different questions at different levels of the architecture. [**Revision: Sec. 3.2, new paragraph after Table 1.**]

Point 6 — "Unified semantics" vs. "hybrid PN" contradiction

> *In the introduction, the author states that their model provides unified semantics, as opposed to a hybrid Petri net approach, and then proceed to define their model as an extension of a hybrid Petri net.*

Correct; this was a real contradiction. The revised manuscript no longer contrasts SHPN with hybrid PN approaches. Instead, contributions point 3 in Section 4.1 now reads: *"SHPN is a hybrid Petri net in the sense of supporting coexistent continuous and stochastic dynamics, but extends classical hybrid PN by adding unified signal-type semantics that resolve the sub-model separation limitation of prior approaches."* The word "unified" now refers to the arc-type semantics being unified within a single arc set, not to a contrast with hybridness. [**Revision: Sec. 4.1, contributions bullet 3.**]

Point 7 — AI generation concern

> *The text itself is rife with poorly defined or outright meaningless phrases with the intention of making it sound "smart" without actually saying anything. A typical artifact of AI generation.*

We acknowledge the concern and have added an explicit AI disclosure to the Acknowledgments section (also described in our Editor response above). The scientific content of the manuscript

— the formalism, the theorems, the biological interpretations, the experimental comparisons — originates entirely from the author's scientific judgement. AI tools assisted with prose editing. We have taken the reviewer's stylistic criticism seriously and revised passages that were identified as vague or overclaimed, in particular the "first- principles" language and the remote sensing section.

Reviewer #3 (anonymous)

> *The manuscript presents an ambitious and potentially valuable formalism, Signal Hierarchical Petri Nets, intended to unify metabolic and regulatory modelling by allowing selected biological molecules to participate simultaneously in mass-transfer reactions and consumptive signal flow arcs. The conceptual distinction between normal arcs, test arcs, and signal flow arcs is interesting, and the idea of using signal-token consumption to represent irreversible biological commitment could be a useful contribution to systems biology and formal modelling. It is a well-written manuscript that clearly outlines the theory and analysis, which support the conclusions drawn.*

We thank Reviewer 3 for this generous assessment and for the positive recommendation. No specific concerns were raised.

Summary of all changes

For convenience, the table below maps each reviewer point to the corresponding change in the revised manuscript.

**Reviewer point**	**Change**	**Location**
Editor: contradictory claims	Replaced all "first-principles" language; corrected hybrid-PN framing	Throughout; Intro; Sec. 4.1
Editor: AI disclosure	Acknowledgments paragraph added	Sec. Acknowledgments
Editor: Table refs	Added in-text `\ref` calls	Sec. 3.2; Sec. 4.4
R1-C1: "first-principles"	Replaced with "topology-driven"	All 10 occurrences
R1-C2: second biological system	Pre-print developmental record cited; B. subtilis remains sole validated system	Sec. 5.3 (new)
R1-C3: acyclicity / feedback	New paragraph + Discussion opening	Sec. 3.2; Sec. 6
R1-C4: PN comparison	New comparison paragraph	Sec. 4.4
R1-minor: dual-graph diagram	Fig. 1 added	End of Introduction
R2-P1: T undefined	"Let T be..." added	Def. 1
R2-P2: $E_s \to F_s$	Global replacement	All occurrences
R2-P3: W_t missing	Convention stated in F_t bullet	Sec. 4.1
R2-P4: Thm. 2/3 missing hypotheses	Hypotheses moved into statement	Theorems 2 and 3
R2-P5: Φ subsumes G_S?	Clarifying paragraph	Sec. 3.2
R2-P6: hybrid-PN contradiction	Contributions bullet 3 rewritten	Sec. 4.1
