# Supplementary material for: Signal hierarchical petri nets: Formal semantics of hierarchical regulatory control of biological systems
Source: PLoS One. 2026 Aug 21;21(8):e0355540. doi: 10.1371/journal.pone.0355540 (PMC13496463; doi:10.1371/journal.pone.0355540)
Supplement: Supplementary file 3 [file pone.0355540.s004.pdf]

Response to Reviewers — Second Revision
Manuscript: PONE-D-26-05900R1
Title: Signal Hierarchical Petri Nets: Formal Semantics of Hierarchical Regulatory Control of Biological Systems
Authors: Eugênio Simão
Date: July 20, 2026

We thank the editor and both reviewers for the second round of evaluation. Reviewer 1 has indicated that all previous concerns have been addressed. Reviewer 2 (Dr. Juri Kolčák) maintains a rejection recommendation and raises a further set of technical points. We respond to each point below with either a correction or a rebuttal, supported in each case by a specific revision reference.

Revision references of the form [**Revision: Sec. X**] indicate where each change appears in the revised manuscript. A track-changes PDF generated by `latexdiff` against the R1 submission is submitted alongside this letter.

Academic Editor (Prof. Karthik Raman)

> *While the reviewers appreciate the contributions, one of them continues to have some concerns, which must be addressed (or rebutted effectively) before accepting the manuscript for publication.*

We have addressed each of Reviewer 2's remaining concerns. Genuine technical errors have been corrected; points that rest on a mischaracterisation of the stochastic Petri net formalism or of contextual Petri net theory have been rebutted with precise citations and arguments. The core results — the acyclicity theorem, the preemption theorem, the basin computability result, and the biological validation — are unaffected by the corrections and are strengthened by the additional clarifications.

Reviewer #1 (anonymous)

> *As far as your theoretical considerations and hypothesis are concerned and supported (corroborated) by your accessible data, the main theoretical conclusions are valid. I think the article has mended all the minor issues I could find. I tend to agree that the major issue pointed out by other referees has been mitigated.*

We thank Reviewer 1 for this positive assessment. No further changes were requested.

Reviewer #2 (Dr. Juri Kolčák)

We thank Dr. Kolčák for the continued and detailed technical engagement. We address his two major comments, followed by every specific point raised, in order.

Major comment (1) — Rate functions Φ absent from enabling/firing semantics

> *It remains a fact that while Ψ (Rate functions) are introduced throughout the paper, they are not used in the semantics of the model, making them completely useless and superfluous.*

This comment conflates the two standard layers of stochastic Petri net semantics, which are architecturally separate by design. In stochastic Petri nets (Goss & Peccoud 1998; Haas 2002), the formalism has two distinct layers:

1. *Structural enabling* (topology-based): the Enabled predicate determines *whether* a transition may fire, based solely on token counts and arc structure. This is the layer the reviewer is examining.
2. *Kinetic propensity* (Φ): the rate function determines *how fast* an enabled transition fires, governing the simulation dynamics (τ-leaping propensity, ODE rates).

Φ is intentionally absent from the Enabled predicate and the Firing rule: this is the standard stochastic PN architecture, not a deficiency. A formalism in which Φ appeared in the enabling condition would conflate qualitative reachability (structural) with quantitative dynamics (kinetic), making structural theorems unprovable without specifying rates— precisely the limitation classical PN theory was designed to avoid.

We have added an explicit paragraph to the Φ/F_s remark ([**Revision: Sec. 4.1, Remark after signal flow arc definition**]) and to the 13-tuple definition ([**Revision: Sec. 4.1, Definition 5**]) making this two-layer architecture explicit and citing the stochastic PN literature.

Major comment (2) — Motivation for test arcs over loops

> *It is well known that test arcs (often called read arcs) create a semantic difference to loops ($W(p,t) = W(t,p)$) when concurrency is a concern, but are essentially indistinguishable from loops in other settings. What is the motivation for inclusion of test arcs into the proposed model, why are loops not enough? Additionally, test arcs could easily be modelled using the "RateFunctions" Ψ as they have been described.*

(a) Semantic distinctness (Contextual Petri Nets): The reviewer cites Baldan, Corradini & Montanari ("Contextual Petri Nets, Asymmetric Event Structures, and Processes") as evidence that test arcs and loops are distinguishable only under concurrency. We note that this reference establishes precisely the opposite of the reviewer's claim: Baldan et al. introduced contextual/read arcs as a formally distinct arc type with different causal semantics from loops, and their paper is the canonical reference for the inclusion of non-consuming observation arcs in Petri net theory. Loops $W(p,t) = W(t,p)$ consume and regenerate the same number of tokens: they contribute to the stoichiometric firing rule $M'(p) = M(p) - W((p,t)) + W((t,p))$, which is zero only because the two terms cancel. Test arcs do not participate in the firing rule at all: $M'(p) = M(p)$ unconditionally. This is a semantic difference in the single-step firing semantics, independent of any concurrent setting.

(b) Biological correctness: Test arcs correctly model biological catalysts: enzymes and co-factors that gate reactions without being consumed must leave the stoichiometric balance

unchanged. Loops $W(p,t) = W(t,p)$ would introduce a spurious consume-then-regenerate cycle in the stoichiometric record — misrepresenting the biology even though the net token change is zero. Test arcs make the read-only character explicit and verifiable in the arc graph, enabling formal proofs that depend on the distinction (e.g., that test arcs cannot create basin boundaries, whereas signal flow arcs can).

We have updated the test arc paragraph with both justifications ([**Revision: Sec. 4.1, Test arc definition**]).

Specific point: Page 10, Theorem 2 proof

> *PreemptionCheck has been included in prerequisites (although it has not been defined yet), but the proof is false. The induction hypothesis is that Enabled is false for any span. The induction step then relies on PreemptionCheck to prove Enabled is also false for span = k, however, Enabled (induction hypothesis) is not an input of PreemptionCheck as it only relies on the other three Enabled components (NormalEnabled, TestEnabled, SignalEnabled).*

The corrected proof proceeds as follows. *Base case* ($k = 1$): $M(p_i) < \theta(t_i)$ implies $M(p_i) < \theta(t_i) + W_s((p_i, t_i))$ (since $W_s > 0$), hence $\neg\text{SignalEnabled}(t_i, M)$. Since $\text{PreemptionCheck}(t_j, M)$ requires $\text{SignalEnabled}(t_i, M)$ for every $t_i \in {}^{\bullet}_{s}p_s$ where $p_s \in {}^{\bullet}_{s}t_j$, we have $\neg\text{PreemptionCheck}(t_j, M)$, hence $\neg\text{Enabled}(t_j, M)$. *Inductive step*: the propagation uses $\neg$SignalEnabled at each layer rather than $\neg$Enabled, tracing through the F_s-acyclic path.

[Revision: Sec. 2.2, Theorem 2, proof rewritten.]

Specific point: Page 11, Theorem 3 proof

> *There is no restriction on transitions in $F \setminus F_s$ producing tokens in p_s. In fact, further in the text the author outright states that the metabolic "horizontal" transitions are allowed to replenish signalling places.*

The theorem statement already carries the hypothesis "assuming no other transition regenerates p_s via F_s" (i.e., no $t' \neq t$ with $(t', p_s) \in F_s$). The reviewer's concern is that transitions in $F \setminus F_s$ (normal arcs) can also produce tokens in p_s. This is correct biologically and is an intentional design: metabolic transitions in G_E can replenish signal places via *normal arcs*, representing ongoing production of the signalling species. The theorem applies to the signal-arc topology only, establishing that no *signal-arc path* returns to p_s after commitment firing. Metabolic replenishment via normal arcs is not a counter-example to the basin partition theorem because it affects G_E dynamics, not the G_s commitment topology.

The text has been clarified to make this scope explicit. **[Revision: Sec. 2.3, Theorem 3, added scope note.]**

Specific point: Page 18, Definition 5 — F_s acyclicity redundancy in equation (8)

> *F_s being acyclic implies $W_s(t,p) = 0$ if $W_s(p,t) > 0$; there is therefore no need to use it in the equation.*

The reviewer is formally correct: for the acyclic case, the backward weight $W_s((t,p))$ is necessarily zero. The general form $M'(p_s) = M(p_s) - W_s((p_s,t)) + W_s((t,p_s))$ is retained

for two reasons: (1) it matches the standard PN firing rule format that readers familiar with the literature will expect; (2) the backward term makes explicit that in the acyclic case $W_s((t, p_s)) = 0$, so $M'(p_s) = M(p_s) - W_s((p_s, t))$, which is informative rather than redundant. No change made; a clarifying remark has been added. **[Revision: Sec. 4.2, Firing Rule remark.]**

Specific point: Page 19, line 442 — $\tau_t = 0$ inconsistency

> *Here $\tau_t = 0$ is used to mean $M(p) > 0$, however, in the definition of the TestPredicate, as well as earlier in the paragraph, the not-sharp inequality $M(p) \geq \tau_t$ is used.*

Fixed: The reviewer is correct. $M(p) \geq 0$ (non-sharp, default $\tau_t = 0$) is vacuously true and does not implement a presence check. The TestEnabled predicate has been corrected to use strict inequality $M(p) > \tau_t((p, t))$ throughout, so that the default $\tau_t = 0$ correctly implements $M(p) > 0$ (presence check). **[Revision: Sec. 4.2, Definition 6, TestEnabled predicate and surrounding remark.]**

Specific point: Page 20, lines 456–461 — commitment states mislabelled

> *The commitment states appear mislabelled: $M(p_s) < M_{\text{commit}}(p_s)$ should be post-commitment ("irreversibly emptied") while $M(p_s) > M_{\text{commit}}(p_s)$ should be pre-commitment ("fireable").*

Fixed: The reviewer identified a genuine ambiguity. Both interpretations are valid but describe different time points:

- *Before firing*: $M(p_s) < M_{\text{commit}}$ means signal not yet at threshold (pre-commitment, transition disabled).
- *After firing*: $M'(p_s) = \theta(t) < M_{\text{commit}}$ means signal consumed (post-commitment, irreversible).

The labelling now explicitly distinguishes these two cases by adding time-point qualifiers "(before firing)" and "(after firing)". **[Revision: Sec. 4.2, Commitment Semantics, enumeration items 1 and 3.]**

Specific point: Page 21, Fig. 2 — caption and threshold crossing

> *None of the dynamics appear to exceed the $\theta_{\sigma H}$ threshold around $t = 300$, which is marked as the "global commitment time".*

The figure shows 50 stochastic replicates. Commitment occurs when any individual trajectory stochastically crosses the threshold; the mean trajectory does *not* cross it (this is the threshold-crossing probability mechanism: $P(\text{spor}) = P(\sigma_H^{\text{stoch}} > \theta_{\sigma H})$). The caption has been revised to state explicitly that commitment is a *stochastic threshold-crossing event*, not a deterministic mean-field crossing, and that the marked time $t_{\text{commit}} \approx 300$ min is the time at which the first sporulating replicate fires T_septation, not the time at which the mean trajectory crosses $\theta_{\sigma H}$. **[Revision: Sec. 5.1, Fig. 2 caption.]**

Specific point: Page 22, Def. 6 — Φ and C not used in Enabled or firing

> *Φ, the untyped kinetic rate laws, are never used in the definition of Enabled or in firing of transitions. Why do they appear in this definition? Similarly, the untyped "C" assigning stoichiometric coefficients is never used in definition of Enabled or in firing.*

As addressed in Major Comment (1) above: Φ governs the propensity layer (simulation dynamics, EPR computation), not the structural enabling layer. This is the standard stochastic PN architecture. C provides stoichiometric coefficients for ODE-mode transitions (continuous flux computation), distinct from discrete token weights W. Both Φ and C have been annotated with explicit simulation-role descriptions in Definition 5. **[Revision: Sec. 4.1, Definition 5.]**

Specific point: Page 25, line 569 — reachability complexity

> *The author claims reachability in Petri nets is EXPSPACE-complete, but the citation provided only claims EXPSPACE-hardness. In fact, reachability in vector addition systems is Ackermann-complete (Czerwiński and Orlikowski).*

Fixed: The reviewer is correct on both counts. The claim has been corrected to *Ackermann-complete* at all five occurrences, and the Czerwiński & Orlikowski (FOCS 2021) reference has been added to the bibliography. **[Revision: Sec. 5.2 proof, Sec. 5.3 discussion, Sec. 6 complexity paragraph, Sec. 7 Table 2, Sec. 7 summary; and bibliography.]**

Specific point: Page 26, line 585 — test arc sharp inequality

> *Test arcs "redefined" as sharp inequality although TestPredicate definition uses $\geq$.*

Fixed: Corrected: the TestEnabled predicate now uses strict inequality $M(p) > \tau_t((p,t))$ consistently throughout, resolving this inconsistency. **[Revision: Same as τ_t fix above.]**

Specific point: Page 30, Equation (15) — M_{commit} trivial

> *The biologically relevant threshold is obviously the input θ; W_s is merely a modeller's choice. The value of M_{commit} is trivial to compute and useless.*

The formula $M_{\text{commit}} = \theta + W_s$ is retained because it serves a concrete predictive purpose in the biological validation. The reviewer's characterisation as "trivial" is perhaps true in the abstract, but the claim of the paper is not the formula itself but the capacity to compute structural commitment thresholds directly from arc-level parameters θ and W_s without simulation or global fitting. For signal-flow arc inputs (e.g., GTP_pool gating $T_{\text{septation}}$), $M_{\text{commit}} = \theta + W_s$ yields the structural threshold from topology alone. We note that $\theta_{\sigma H} = 1.60\,\mu\text{M}$ is an *effective kinetic threshold* identified via simulation (Sweep A), not a structural M_{commit} computation, since σ_H gates $T_{\text{septation}}$ through the rate function Φ (remote sensing) rather than a signal-flow arc. The manuscript has been updated to make this distinction explicit. A sentence clarifying the predictive role of the structural formula has been added. **[Revision: Sec. 4.3.]**

Specific point: Page 31, Theorem 6.1.1 — proof "hardly necessary"

> *This proof is hardly necessary, given that the layer assignment is indeed just a topological sorting.*

Agreed that topological sorting is well-known. The theorem and algorithm are retained because they serve a different purpose: they provide a *constructive failure condition* (the algorithm returns FAIL if G_s contains a cycle), which is the computational mechanism for enforcing the Acyclicity Requirement of Theorem 1 during model construction. A sentence has been added making this purpose explicit. [**Revision: Sec. 4.4.**]

Specific point: Page 32, Proposition 3 — copies Theorem 2

> *This proposition is effectively a copy of Theorem 2 (page 10), providing no further insights. The proof is also wrong, same as Theorem 2.*

Proposition 3 applies the general Hierarchical Preemption result (Theorem 2) to the specific case of the *B. subtilis* model introduced in the validation section, making the cascade hierarchy concrete. The proof has been replaced with a direct reference to Theorem 2 rather than repeating the general argument, and the proposition is now labelled "Corollary (Biological Instantiation of Theorem 2)" to make the derivation relation explicit. [**Revision: Sec. 5.2.**]

Specific point: Page 33, Proposition 4 — trivial $O(1)$ computation

> *The fact that sum of two input constants is computable in $O(1)$ is trivial and requires no proof.*

Agreed. The "proof" has been removed; the proposition is retained as a remark stating the $O(1)$ threshold formula, since its biological significance (direct computation from literature kinetic constants with no simulation) justifies its explicit statement even if the arithmetic is elementary. [**Revision: Sec. 5.2.**]

Specific point: Figure 3 — signal arcs form loops

> *Although acyclicity requirement of signal arcs is stressed as a strong constraint, the signal arcs in Figure 3 immediately form loops. See for instance KinA_P $\rightarrow$ T_Spo0F_phosphorylation $\rightarrow$ KinA_kinase3.0 $\rightarrow$ T_kinA_activation.*

All four arcs have been corrected to type `normal` in the model file. Cycle detection re-run on the corrected model confirms G_s is acyclic ($|\Psi| = 20$ signal-flow arcs remaining, zero cycles). Figure 3 has been regenerated from the corrected model. [**Revision: Model file `bacillus_sporulation_v9.shy` (4 arc types corrected); Fig. 3 regenerated; model description in Sec. 5.1 updated.**]

Summary of all R2 changes

R2 point	Action	Location
Fig. 3 signal arc cycles	Fixed: 4 arcs reclassified to normal; G_s verified acyclic	Model file; Fig. 3; Sec. 5.1
Theorem 2 proof	Fixed: proof now uses ¬SignalEnabled, not ¬Enabled	Sec. 2.2, Thm. 2 proof
τ_t / test arc inconsistency	Fixed: TestEnabled changed to strict $>$ throughout	Sec. 4.2, Def. 6
Commitment labelling ambiguity	Fixed: time-point qualifiers added	Sec. 4.2, Commitment Semantics
Reachability EXPSPACE claim	Fixed: Ackermann-complete at 5 locations	Sec. 5.2, 5.3, 6, 7
Φ not in Enabled/Firing	Rebuttal: standard stochastic PN two-layer architecture	Sec. 4.1, Def. 5 remark
Test arcs vs. loops	Rebuttal: Baldan et al. (his own citation) + biological correctness argument	Sec. 4.1, test arc definition
Def. 6 Φ, C role	Clarified: simulation propensity / ODE stoichiometry	Sec. 4.1, Def. 5
Theorem 3 scope	Clarified: applies to G_s, not to G_E normal arcs	Sec. 2.3, Thm. 3
Fig. 2 threshold crossing	Clarified: stochastic crossing, not mean-field	Sec. 5.1, Fig. 2 caption
M_{commit} triviality	Rebuttal: predictive role from kinetic constants	Sec. 4.3
Thm. 6.1.1 topological sort	Clarified: constructive failure condition	Sec. 4.4
Prop. 3 copies Thm. 2	Restructured as Corollary; proof replaced with reference	Sec. 5.2
Prop. 4 trivial	Proof removed; retained as remark	Sec. 5.2
