# Supplementary material for: Signal hierarchical petri nets: Formal semantics of hierarchical regulatory control of biological systems
Source: PLoS One. 2026 Aug 21;21(8):e0355540. doi: 10.1371/journal.pone.0355540 (PMC13496463; doi:10.1371/journal.pone.0355540)
Supplement: Peer review history

# Author comment: Author response to Decision Letter 0

**DOI:** 10.1371/journal.pone.0355540.r001

**Transfer Alert**

This paper was transferred from another journal. As a result, its full editorial history (including decision letters, peer reviews and author responses) may not be present.

3 Feb 2026

# Peer review: Decision Letter 0

**DOI:** 10.1371/journal.pone.0355540.r002

**Authors:** Jianhong Zhou

17 Jun 2026

Semantics of Hierarchical Regulatory Control

Dear Dr. Simao,

Please submit your revised manuscript by Aug 01 2026 11:59PM. If you will need more time than this to complete your revisions, please reply to this message or contact the journal office at plosone@plos.org . When you're ready to submit your revision, log on to https://www.editorialmanager.com/pone/ and select the 'Submissions Needing Revision' folder to locate your manuscript file.

- A letter that responds to each point raised by the academic editor and reviewer(s). You should upload this letter as a separate file labeled 'Response to Reviewers'.
- A marked-up copy of your manuscript that highlights changes made to the original version. You should upload this as a separate file labeled 'Revised Manuscript with Track Changes'.
- An unmarked version of your revised paper without tracked changes. You should upload this as a separate file labeled 'Manuscript'.

If you would like to make changes to your financial disclosure, please include your updated statement in your cover letter. Guidelines for resubmitting your figure files are available below the reviewer comments at the end of this letter.

If applicable, we recommend that you deposit your laboratory protocols in protocols.io to enhance the reproducibility of your results. Protocols.io assigns your protocol its own identifier (DOI) so that it can be cited independently in the future. For instructions see: https://journals.plos.org/plosone/s/submission-guidelines#loc-laboratory-protocols . Additionally, PLOS ONE offers an option for publishing peer-reviewed Lab Protocol articles, which describe protocols hosted on protocols.io. Read more information on sharing protocols at https://plos.org/protocols?utm_medium=editorial-email&utm_source=authorletters&utm_campaign=protocols .

As the corresponding author, your ORCID iD is verified in the submission system and will appear in the published article. PLOS supports the use of ORCID, and we encourage all coauthors to register for an ORCID iD and use it as well. Please encourage your coauthors to verify their ORCID iD within the submission system before final acceptance, as unverified ORCID iDs will not appear in the published article. *Only*   the individual author can complete the verification step; PLOS staff *cannot*   verify ORCID iDs on behalf of authors.

We look forward to receiving your revised manuscript.

Kind regards,

Jianhong Zhou

Staff Editor

PLOS One

Journal Requirements:

When submitting your revision, we need you to address these additional requirements.

1.Please ensure that your manuscript meets PLOS ONE's style requirements, including those for file naming. The PLOS ONE style templates can be found at

https://journals.plos.org/plosone/s/file?id=wjVg/PLOSOne_formatting_sample_main_body.pdf and

https://journals.plos.org/plosone/s/file?id=ba62/PLOSOne_formatting_sample_title_authors_affiliations.pdf

2. When completing the data availability statement of the submission form, you indicated that you will make your data available on acceptance. We strongly recommend all authors decide on a data sharing plan before acceptance, as the process can be lengthy and hold up publication timelines. Please note that, though access restrictions are acceptable now, your entire data will need to be made freely accessible if your manuscript is accepted for publication. This policy applies to all data except where public deposition would breach compliance with the protocol approved by your research ethics board. If you are unable to adhere to our open data policy, please kindly revise your statement to explain your reasoning and we will seek the editor's input on an exemption. Please be assured that, once you have provided your new statement, the assessment of your exemption will not hold up the peer review process.

3. We note you have included a table to which you do not refer in the text of your manuscript. Please ensure that you refer to Table 1 and 2 in your text; if accepted, production will need this reference to link the reader to the Table.

4. If the reviewer comments include a recommendation to cite specific previously published works, please review and evaluate these publications to determine whether they are relevant and should be cited. There is no requirement to cite these works unless the editor has indicated otherwise.

Additional Editor Comments:

The manuscript has been evaluated by three reviewers, and their comments are available below.

The reviewers have raised a number of concerns that need attention, especially the contradictory claims. In addition, please disclose if there is any artificial intelligence (AI) tools and technologies usage following our policy (https://journals.plos.org/plosone/s/ethical-publishing-practice#loc-artificial-intelligence-tools-and-technologies).

[Note: HTML markup is below. Please do not edit.]

Reviewer's Responses to Questions

**Comments to the Author**

1. Is the manuscript technically sound, and do the data support the conclusions?

Reviewer #1: Partly

Reviewer #2: No

Reviewer #3: Yes

**********

2. Has the statistical analysis been performed appropriately and rigorously?

Reviewer #1: N/A

Reviewer #2: N/A

Reviewer #3: Yes

**********

3. Have the authors made all data underlying the findings in their manuscript fully available?

The PLOS Data policy

Reviewer #1: Yes

Reviewer #2: Yes

Reviewer #3: Yes

**********

4. Is the manuscript presented in an intelligible fashion and written in standard English?

Reviewer #1: Yes

Reviewer #2: No

Reviewer #3: Yes

**********

Reviewer #1: Summary

This manuscript introduces \emph{Signal Hierarchical Petri Nets} (SHPN), a 13-tuple extension of classical Biological Petri Nets grounded in what the author terms \emph{Signal Hierarchy Theory}. The central idea is to distinguish between horizontal mass-transfer arcs and vertical consumptive signal-flow arcs, thereby enabling unified metabolic–regulatory modeling within a single formalism. The framework establishes structural theorems (acyclicity, hierarchical preemption, irreversible basin partitioning) and proposes that biological commitment thresholds can be computed structurally via the closed-form expression

\[

M_{\mathrm{commit}} = \theta(t) + W_s((p_s,t)).

\]

The approach is illustrated through a case study of \emph{Bacillus subtilis} sporulation, where the predicted ATP commitment threshold closely approximates experimental values.

The rigor is very good. However, the theorems are what we should expect from the definitions. They have the main purpose of justifying the definitions, being a kind of mathematical retionale. This is well-done.

\subsection*{Major Strengths}

\begin{enumerate}

\item \textbf{Conceptual originality.}

The introduction of consumptive signal semantics represents a clear conceptual advance beyond classical Bio-PN and test-arc extensions. The distinction between mass transfer and signal consumption is well articulated and mathematically precise.

\item \textbf{Formal rigor.}

The manuscript provides explicit definitions, enablement predicates, firing rules, and structural theorems. The acyclicity and hierarchical preemption results are logically coherent and appropriately proven.

\item \textbf{Unified modeling of metabolism and regulation.}

Allowing the same place to participate in both metabolic and regulatory roles through arc-type assignment is biologically motivated and technically elegant. This avoids artificial duplication of species often seen in hybrid approaches.

\item \textbf{Closed-form threshold computation.}

The structural commitment formula is simple, interpretable, and potentially impactful. Turning commitment thresholds into computable topological properties is a noteworthy contribution.

\item \textbf{Illustrative biological validation.}

The \emph{B. subtilis} sporulation example is carefully developed and includes sensitivity analysis, strengthening the plausibility of the framework.

\end{enumerate}

\subsection*{Major Concerns}

\begin{enumerate}

\item \textbf{Scope of ``first-principles'' claim.}

While thresholds are computed structurally, the parameters $\theta(t)$ and $W_s$ are derived from experimentally measured biochemical constants. The manuscript would benefit from clarifying that the prediction is topology-driven but parameter-dependent, rather than entirely parameter-free.

\item \textbf{Biological generality.}

The main detailed validation focuses on a single biological system. Although additional examples are mentioned, they are not fully developed. For a generalist journal such as \emph{PLOS ONE}, broader empirical validation or computational benchmarking across multiple systems would strengthen the manuscript.

\item \textbf{Acyclicity assumption.}

The requirement that signal flow graphs be acyclic is theoretically justified, but many biological regulatory networks exhibit feedback loops. The manuscript addresses this through remote sensing via rate functions $\Phi$, yet the conceptual separation between ``connected'' and ``remote'' regulation may require further clarification to avoid the impression of biological oversimplification.

\item \textbf{Comparison with existing extended PN frameworks.}

A more systematic comparison with alternative Petri net extensions (e.g., inhibitor arcs, colored PN, hybrid PN variants) would help situate the contribution within the broader modeling literature. There are approaches defined on top of universal semantic principles, using Petri-Nets, that models the BioMolecular realm as a Turing Machine models the symbolic computational realm, via Turing-Church Thesis (Hypothesis). They do not separate signals and mass, so that a bit more of fundamental explaining would be welcome, since they catch the Intensional Semantics of the BioMolecular processes, without needing external paprameters.

\end{enumerate}

\subsection*{Minor Comments}

\begin{itemize}

\item The terminology is dense and layered. A high-level schematic diagram early in the manuscript summarizing the dual-graph architecture would improve accessibility.

\item Some sections could be streamlined to reduce repetition between theoretical exposition and biological discussion.

\end{itemize}

\subsection*{Recommendation}

This manuscript presents a mathematically rigorous and conceptually innovative extension of Biological Petri Nets with potential relevance for systems biology modeling. The theoretical contribution is substantial and internally consistent. However, clarification of the ``first-principles'' claim and expanded biological validation would significantly strengthen the work for publication in \emph{PLOS ONE}.

Subject to satisfactory revision addressing the concerns above, I consider the manuscript suitable for publication. The edition and presentation is ok. However, the author should observe our observations and suggestions on the content and foundational principles.

Reviewer #2: I recommend the article to be rejected, due to poor formal foundations which in some cases make it impossible to verify the validity of the presented claims, as well as outright contradictory claims.

I will keep this short and skip any comments on the introduction, and instead highlight glaring deficiences in the subsequent sections, beginning with Section 2.

Section 2.1 opens with "formal" definitions, however, immediately relies on undefined conceps (T has not been defined in Def. 1), disrespects its own established notation (E_s magically turns into F_s) and it immediately becomes unclear what is the intent or purpose of these constructions.

The Theorems following in Section 2.2 are either trivial or plain wrong, depending on how one "completes" the definitions. While the "Acyclicity Requirement" is a trivial conclusion assuming the definition are completed in the shape of a standard Petri net, the "Hierarchical Preemption" requires extensive constrains on the shape of the undefined set of transitions T, which have never been made, nor alluded to, making it incorrect or at least superfluous. Essentially the same claim is made in Theorem 3, where the "irreversibility" part of the proof, which relies on those missing constraints, is simply hand-waved away.

The same trend of incomplete and disconnected "formal" claims continues through the rest of the paper.

Among others, I have found the following claims and constructions quite astonishing:

In several places, the "model" is advertised as capable of computing regulation thresholds from network topology, while in the actual definition and the adjoint "case study", it is admitted these thresholds are parameters of the model, which is entirely unsurprising.

In section 3.2, "remote sensing" is introduced in which transitions are assigned a function that determines whether they are enabled without any regards to the network topology or constraints on the shape of the function itself. This formalism is far more rich than a classical Petri net, and makes literally all other formalisms discussed in the paper obsolete, as it subsumes them. It is also, not novel. It is never properly explained how these functions are actually used, meaning they either make the rest of the paper obsolete, or, are in fact not used at all.

When the main model is finally introduced "formally" (section 4.1) it does not even include all the components utilised in prior text (e.g. W_t). Moreover, in the introdution, the author states that their model provides unified semantics, as opposed to a hybrid Petri Net approach, and then proceed to define their model as an extension of a hybrid Petri Net.

Altogether, making out the exact formalism out of the article is impossible. The vague idea of the intention paints a picture of a model that does not introduce anything new, and is in fact at best a special case of classical Petri net or hybrid Petri net models employed for the same purpose, restricting them to applications in which the regulatory component is known to be acyclic.

Finally, the text itself is rife with poorly defined or outright meaningless phrases with the intention of making it sound "smart" without actually saying anything. A typical artifact of AI generation.

Reviewer #3: The manuscript presents an ambitious and potentially valuable formalism, Signal Hierarchical Petri Nets, intended to unify metabolic and regulatory modelling by allowing selected biological molecules to participate simultaneously in mass-transfer reactions and consumptive signal flow arcs. The conceptual distinction between normal arcs, test arcs, and signal flow arcs is interesting, and the idea of using signal-token consumption to represent irreversible biological commitment could be a useful contribution to systems biology and formal modelling. It is a well-written manuscript that clearly outlines the theory and analysis, which support the conclusions drawn.

**********

what does this mean? ). If published, this will include your full peer review and any attached files.

If you choose “no”, your identity will remain anonymous but your review may still be made public.

**Do you want your identity to be public for this peer review?**   For information about this choice, including consent withdrawal, please see our Privacy Policy

Reviewer #1: **Yes:**  Prof. Edward Hermann Haeusler

Reviewer #2: **Yes:**  Juri Kolčák

Reviewer #3: No

**********

[NOTE: If reviewer comments were submitted as an attachment file, they will be attached to this email and accessible via the submission site. Please log into your account, locate the manuscript record, and check for the action link "View Attachments". If this link does not appear, there are no attachment files.]

To ensure your figures meet our technical requirements, please review our figure guidelines: https://journals.plos.org/plosone/s/figures

You may also use PLOS’s free figure tool, NAAS, to help you prepare publication quality figures: https://journals.plos.org/plosone/s/figures#loc-tools-for-figure-preparation.

NAAS will assess whether your figures meet our technical requirements by comparing each figure against our figure specifications.

Attachment Submitted filename: PLOSONEReview.pdf

# Author comment: Author response to Decision Letter 1

**DOI:** 10.1371/journal.pone.0355540.r003

30 Jun 2026

Dear Dr. Zhou, Prof. Haeusler, Dr. Kolčák, and Reviewer 3,

Thank you sincerely for the time and expertise each of you brought to evaluating this manuscript. The reviewers' reports were precise, substantive, and genuinely improved the work — both in formal rigour and in its clarity for a broad audience.

A full point-by-point response to every comment raised is provided in the attached Response to Reviewers letter. A cover letter outlining the overall revision strategy is also enclosed, together with a track-changes PDF to facilitate verification.

I hope the revised manuscript reflects the care with which each concern was taken. I remain available to clarify or expand any point further, and I look forward to the editorial decision.

With appreciation and respect,

Simão Eugênio

Universidade Federal de Santa Catarina

Attachment Submitted filename: response_letter.pdf

# Peer review: Decision Letter 1

**DOI:** 10.1371/journal.pone.0355540.r004

**Authors:** Karthik Raman

16 Jul 2026

Dear Dr. Simao,

Please submit your revised manuscript by Aug 30 2026 11:59PM. If you will need more time than this to complete your revisions, please reply to this message or contact the journal office at plosone@plos.org . When you're ready to submit your revision, log on to https://www.editorialmanager.com/pone/ and select the 'Submissions Needing Revision' folder to locate your manuscript file.

- A letter that responds to each point raised by the academic editor and reviewer(s). You should upload this letter as a separate file labeled 'Response to Reviewers'.
- A marked-up copy of your manuscript that highlights changes made to the original version. You should upload this as a separate file labeled 'Revised Manuscript with Track Changes'.
- An unmarked version of your revised paper without tracked changes. You should upload this as a separate file labeled 'Manuscript'.

If you would like to make changes to your financial disclosure, please include your updated statement in your cover letter. Guidelines for resubmitting your figure files are available below the reviewer comments at the end of this letter.

If applicable, we recommend that you deposit your laboratory protocols in protocols.io to enhance the reproducibility of your results. Protocols.io assigns your protocol its own identifier (DOI) so that it can be cited independently in the future. For instructions see: https://journals.plos.org/plosone/s/submission-guidelines#loc-laboratory-protocols . Additionally, PLOS ONE offers an option for publishing peer-reviewed Lab Protocol articles, which describe protocols hosted on protocols.io. Read more information on sharing protocols at https://plos.org/protocols?utm_medium=editorial-email&utm_source=authorletters&utm_campaign=protocols .

As the corresponding author, your ORCID iD is verified in the submission system and will appear in the published article. PLOS supports the use of ORCID, and we encourage all coauthors to register for an ORCID iD and use it as well. Please encourage your coauthors to verify their ORCID iD within the submission system before final acceptance, as unverified ORCID iDs will not appear in the published article. *Only*   the individual author can complete the verification step; PLOS staff *cannot*   verify ORCID iDs on behalf of authors.

We look forward to receiving your revised manuscript.

Kind regards,

Karthik Raman, Ph.D.

Academic Editor

PLOS One

Journal Requirements:

If the reviewer comments include a recommendation to cite specific previously published works, please review and evaluate these publications to determine whether they are relevant and should be cited. There is no requirement to cite these works unless the editor has indicated otherwise.

Please review your reference list to ensure that it is complete and correct. If you have cited papers that have been retracted, please include the rationale for doing so in the manuscript text, or remove these references and replace them with relevant current references. Any changes to the reference list should be mentioned in the rebuttal letter that accompanies your revised manuscript. If you need to cite a retracted article, indicate the article’s retracted status in the References list and also include a citation and full reference for the retraction notice.

[Note: HTML markup is below. Please do not edit.]

Reviewers' comments:

Reviewer's Responses to Questions

**Comments to the Author**

Reviewer #1: All comments have been addressed

Reviewer #2: (No Response)

**********

2. Is the manuscript technically sound, and do the data support the conclusions?

Reviewer #1: Yes

Reviewer #2: No

**********

3. Has the statistical analysis been performed appropriately and rigorously?

Reviewer #1: N/A

Reviewer #2: N/A

**********

4. Have the authors made all data underlying the findings in their manuscript fully available?

The PLOS Data policy

Reviewer #1: Yes

Reviewer #2: Yes

**********

5. Is the manuscript presented in an intelligible fashion and written in standard English?

Reviewer #1: Yes

Reviewer #2: Yes

**********

Reviewer #1: As far as your theoretical considerations and hypothesis are concerned and supported (corroborated) by your accessible data, the main theoretical conclusions are valid. I think the article has mended all the minor issues I could find. I tend to agree that the major issue pointed out by other referees has been mitigated .

Reviewer #2: I commend the author on going through with the revision, however, my stance remains a strong rejection recommendation.

Overall, the paper lacks tangible or meaningful results or contributions. The described model is bloated, but the paper fails to motivate, in some cases even use, all the components.

Definitions, theorems and in-text claims are continuously repeated. The provided case study is presented in a chaotic manner, the model shown in Figure 3 breaks all principles described in the paper, rendering it effectively useless.

I have two larger comments about the technical constructions presented within the article, for which I simply cannot find any justification:

(1) It remains a fact that while \Psi (Rate functions) are introduced throughout the paper, they are not used in the semantics of the model, making them completely useless and superflous. Intentionally bloating and obfuscating the paper to detract from lack of meaningful content.

(2) It is well known that test arcs (often called read arcs) create a semantic difference to loops (W(p,t)=W(t,p)) when concurrency is a concern (see e.g. [Contextual Petri Nets, Asymmetric Event Structures, and Processes by Paolo Baldan, Andrea Corradini and Ugo Montanari]), however, are essentially indistinguishable from loops in other settings. What is the motivation for inclusion of test arcs into the proposed model, why are loops not enough?

Additionally, test arcs could easily be modelled using the "RateFunctions" \Psi as they have been described, although it is difficult to talk about semantic meaning of RateFunctions since they do not, according to the definition provided, have an impact on enabling or firing of transitions.

What is the motivation for having both test arcs and "RateFunctions"? What does inclusion of test arcs add on top of RateFunctions.

Finally, I offer a list of smaller comments highlighting some deficiencies that motivated my overall summary and rejection recommendation:

Page 10, Theorem 2:

PreemptionCheck has been included in prerequisities (although it has not been defined yet), but the proof is false.

The induction hypotheses is that Enabled is false for any span <k.<br>The induction step then relies on PreemptionCheck to prove Enabled is also false for span =k, however, Enabled (induction hypothesis) is not an input of PreemptionCheck as it only relies on the other three Enabled components (NormalEnabled, TestEnabled, SignalEnabled). Indeed, not Enabled does not carry through layers.

Page 11, Theorem 3:

Although Existence of no t' =/= t \in F_s producting tokens in p_s has been included as a requirement. The proof is still false.

There is no restriction on transitions in F\F_s producing tokents in p_s. In fact, further in the text the author outright states that the metabollic "horizontal" transitions are allowed to replenish signaling places, meaning that any "commited" place can be refilled whenever. E.g. caption of Figure 2 suggests a signalling place being refilled.

Page 18, Definition 5:

F_s being acyclic implies $W_s(t,p)=0$ if $W_s(p,t)>0$, there is therefore no need to use it in the equation (8).

Page 19, line 442:

Here $\tau_t=0$ is used to mean $M(p)>0$, however, in the definition of the TestPredicate, as well as earlier in the paragraph, the not-sharp inequality $M(p)>=\tau_t$ is used.

Page 19, line 446:

F_s being acyclic implies $W_s(t,p)=0$ if $W_s(p,t)>0$, there is therefore no need to use it in the formula.

Page 20, lines 456-461:

The commitment states appear mislabelled: M(p_s) < M_commit(p_s) should be post-commitment ("irreversibly emptied") while M(p_s) > M_commit(p_s) should be pre-commitment ("fireable")

The mislabelling is underlined by "Commitment boundary" claiming to create irreversibility, thus firing at M(p_s)=M_commit(p_s) would lead to pre-commitment state according to the labelling.

Page 21, Fig. 2:

The caption of this figure fails to explain the depicted dynamics.

None of the dynamics appear to exceed the \theta_\sigmaH threshold in the graph, definitely not around t=300, which is marked as the "global commitment time across all 50 stochastic replicates".

The text further claims that t<240 is pre-commitment as M(\sigma_H)<\theta_\sigmaH, however, as mentioned, this same condition appears to hold even beyond t=240.

The text also claims that at t<240 PreemptionCheck disables "downstream layers" irrespective of Spo0A~P abundance, however this abundance appears to be invariant at t<240.

Finally, the caption seems to hint at a difference in variance of Spo0A~P between the sporulating and not-sporulating replicates (assumed red and grey, respectively), however, the figure offers no visible difference.

Overall, I believe inclusion of the utilised net structure at this point would greatly aid undestanding of the modelled system and the displayed behaviour, as it is unclear which layers are downstream from \sigma_H or what is the "T_septation" transition(?).

Page 22, Def. 6:

\Phi, the untyped kinetic rate laws are never used in the definition of Enabled or in firing of transitions. Why do they appear in this definition?

Similarly, the untyped "C" assigning soichiometric coefficients (how does this differ from the arc weights in W?) is never used in definition of Enabled or in firing. What is the use?

Page 23, lines 527-532:

An attempt at biological illustration of remote information transfer is made, however, as \Phi does not appear in transition enabling or firing, it is impossible to discern it's meaning.

Page 23, line 533:

This paragraph repeats what has already been stated on page 19, line 445, uselessly bloating and obfuscating the paper.

Page 25, line 569:

The author claims reachability in Petri nets is EXPSPACE-complete, but the citation provided (which is malformed, Mogens Nielson is not a co-author of this work) only claims EXPSPACE-hardness.

In fact, in [Reachability in Vector Addition Systems is Ackermann-complete by Wojciech Czerwiński and Łukasz Orlikowski] the authors prove reachability in vector addition systems (into which Petri nets lineary reduce) is Ackermann-complete.

Page 26, line 585:

Test arcs "redefined" as sharp inequality although TestPredicate definition uses >=

Page 27, lines 597-620:

Largely just reiteration of what has already been elaborated on page 23, lines 508-518, uselessly bloating the article.

Page 30, Equation (15):

The article fails to explain why this is in any way a relevant result.

The biologically relevant and interesting threshold is obviously the input \theta, W_s((p_s,t)) is merely a modellers choice. The value of M_commit(p_s) is then not only trivial to compute, but also useless, as it simply reiterates the already known biological threshold \theta ammended with a constant chosen by the modeller.

Page 31, Theorem 6.1.1:

This proof is hardly necessary, (as is explicit statement of the algorithm), given that the layer assignment is indeed just a Topological sorting, for which algorithms are well known and described.

While it is not inherently incorrect to include the algorithm and the proof, it largely amounts to just bloating an already unnecessarily long work.

Page 32, Proposition 3:

This proposition is effectively a copy Theorem 2 (page 10), providing no further insights.

The proof is also wrong, same as Theorem 2.

Page 33, Proposition 4:

The fact that sum of two input constants is computable in O(1) is trivial and requires no proof. Also constitutes no result.

Page 37, Fig. 3:

This figure immediately underlines the lack of consistency across the paper. Although acyclicity requirement of signal arcs is stressed as a strong constraint on the model (which is supposedly benefitial and biologically relevant), the signal arcs in Figure 3 immediately form loops.

See for instance KinA_P -> T_Spo0F_phosphorylation -> KinA_kinase3.0 -> T_kinA_activation.</k.<br>

**********

what does this mean? ). If published, this will include your full peer review and any attached files.

If you choose “no”, your identity will remain anonymous but your review may still be made public.

**Do you want your identity to be public for this peer review?**   For information about this choice, including consent withdrawal, please see our Privacy Policy

Reviewer #1: No

Reviewer #2: **Yes:**  Juri Kolčák

**********

[NOTE: If reviewer comments were submitted as an attachment file, they will be attached to this email and accessible via the submission site. Please log into your account, locate the manuscript record, and check for the action link "View Attachments". If this link does not appear, there are no attachment files.]

To ensure your figures meet our technical requirements, please review our figure guidelines: https://journals.plos.org/plosone/s/figures

You may also use PLOS’s free figure tool, NAAS, to help you prepare publication quality figures: https://journals.plos.org/plosone/s/figures#loc-tools-for-figure-preparation.

NAAS will assess whether your figures meet our technical requirements by comparing each figure against our figure specifications.

# Author comment: Author response to Decision Letter 2

**DOI:** 10.1371/journal.pone.0355540.r005

22 Jul 2026

Dear Reviewers,

Thank you for your continued engagement with this manuscript. Please find below a summary of the changes made in this second revision.

Reviewer 1: Thank you for confirming that all previous concerns were fully addressed. No further changes were requested, and your assessment is greatly appreciated.

Reviewer 2: Thank you for the detailed technical critique. All genuine errors identified in the first revision have been corrected:

— The most significant correction addresses the G_s cycle violations in Figure 3: four enzyme-regeneration arcs were reclassified from signal-flow to normal type, eliminating all three G_s cycles and restoring full consistency with the paper's acyclicity theorem. The corrected model was re-validated on the GPU server (run_20260719_170034) and deposited on Zenodo (v2.7.1, DOI: 10.5281/zenodo.21478129); results are numerically identical.

— The Theorem 2 (Hierarchical Preemption) proof was rewritten to use negSignalEnabled directly rather than incorrectly propagating negEnabled through PreemptionCheck.

— The TestEnabled predicate was corrected to use strict inequality (M(p) > tau_t) consistent with the default tau_t = 0 presence-check semantics.

— The commitment state labelling was clarified with explicit time-point qualifiers.

— The reachability complexity claim was corrected to Ackermann-complete (Czerwinski & Orlikowski, FOCS 2021).

For the two major comments, the responses are:

(1) Rate functions Phi do not appear in the enabling predicate by design — this is the standard two-layer architecture of stochastic Petri nets (structural enabling vs. kinetic propensity), as described in Goss & Peccoud (1998).

(2) Test arcs are semantically distinct from loops in the single-step firing semantics regardless of concurrency: test arcs leave the marking unconditionally unchanged, while loops consume and regenerate tokens. This is the foundational result of Baldan, Corradini & Montanari (the reviewer's own citation).

A complete point-by-point response to all 14 raised points is provided in the Response to Reviewers document.

Sincerely,

Simão Eugênio

Universidade Federal de Santa Catarina (UFSC), Brazil

Attachment Submitted filename: response_letter_r2.pdf

# Editor report: Decision Letter 2

**DOI:** 10.1371/journal.pone.0355540.r006

**Authors:** Karthik Raman

23 Jul 2026

Signal Hierarchical Petri Nets: Formal

Semantics of Hierarchical Regulatory Control

of Biological Systems

PONE-D-26-05900R2

Dear Dr. Simao,

We’re pleased to inform you that your manuscript has been judged scientifically suitable for publication and will be formally accepted for publication once it meets all outstanding technical requirements.

Within one week, you’ll receive an e-mail detailing the required amendments. When these have been addressed, you’ll receive a formal acceptance letter and your manuscript will be scheduled for publication.

An invoice will be generated when your article is formally accepted. Please note, if your institution has a publishing partnership with PLOS and your article meets the relevant criteria, all or part of your publication costs will be covered. Please make sure your user information is up-to-date by logging into Editorial Manager at Editorial Manager®  and clicking the ‘Update My Information' link at the top of the page. For questions related to billing, please contact billing support .

If your institution or institutions have a press office, please notify them about your upcoming paper to help maximize its impact. If they’ll be preparing press materials, please inform our press team as soon as possible -- no later than 48 hours after receiving the formal acceptance. Your manuscript will remain under strict press embargo until 2 pm Eastern Time on the date of publication. For more information, please contact onepress@plos.org.

Kind regards,

Karthik Raman, Ph.D.

Academic Editor

PLOS One

# Editor report: Acceptance letter

**DOI:** 10.1371/journal.pone.0355540.r007

**Authors:** Karthik Raman

PONE-D-26-05900R2

PLOS One

Dear Dr. Simao,

I'm pleased to inform you that your manuscript has been deemed suitable for publication in PLOS One. Congratulations! Your manuscript is now being handed over to our production team.

At this stage, our production department will prepare your paper for publication. This includes ensuring the following:

* All references, tables, and figures are properly cited

* All relevant supporting information is included in the manuscript submission,

* There are no issues that prevent the paper from being properly typeset

You will receive further instructions from the production team, including instructions on how to review your proof when it is ready. Please keep in mind that we are working through a large volume of accepted articles, so please give us a few days to review your paper and let you know the next and final steps.

Lastly, if your institution or institutions have a press office, please let them know about your upcoming paper now to help maximize its impact. If they'll be preparing press materials, please inform our press team within the next 48 hours. Your manuscript will remain under strict press embargo until 2 pm Eastern Time on the date of publication. For more information, please contact onepress@plos.org.

You will receive an invoice from PLOS for your publication fee after your manuscript has reached the completed accept phase. If you receive an email requesting payment before acceptance or for any other service, this may be a phishing scheme. Learn how to identify phishing emails and protect your accounts at https://explore.plos.org/phishing.

If we can help with anything else, please email us at customercare@plos.org.

Thank you for submitting your work to PLOS ONE and supporting open access.

Kind regards,

PLOS ONE Editorial Office Staff

on behalf of

Dr. Karthik Raman

Academic Editor

PLOS One
